# Rapid Discrimination of *Haemophilus influenzae*, *H. parainfluenzae*, and *H. haemolyticus* by Fluorescence *In Situ* Hybridization (FISH) and Two Matrix-Assisted Laser-Desorption-Ionization Time-of-Flight Mass Spectrometry (MALDI-TOF-MS) Platforms

**DOI:** 10.1371/journal.pone.0063222

**Published:** 2013-04-30

**Authors:** Hagen Frickmann, Martin Christner, Martina Donat, Anja Berger, Andreas Essig, Andreas Podbielski, Ralf Matthias Hagen, Sven Poppert

**Affiliations:** 1 Department of Tropical Medicine at the Bernhard Nocht Institute, German Armed Forces Hospital of Hamburg, Hamburg, Germany; 2 Institute for Medical Microbiology, Virology and Hygiene, University of Rostock, Rostock, Germany; 3 Institute for Medical Microbiology, Virology and Hygiene, University Hospital Eppendorf, Hamburg, Germany; 4 Bavarian Food and Health Authority, Oberschleissheim, Germany; 5 Institute for Medical Microbiology and Hygiene, University of Ulm, Ulm, Germany; 6 Bernhard Nocht Institute for Tropical Medicine, Hamburg, Germany; George Mason University, United States of America

## Abstract

**Background:**

Due to considerable differences in pathogenicity, *Haemophilus influenzae*, *H. parainfluenzae* and *H. haemolyticus* have to be reliably discriminated in routine diagnostics. Retrospective analyses suggest frequent misidentifications of commensal *H. haemolyticus* as *H. influenzae*. In a multi-center approach, we assessed the suitability of fluorescence *in situ* hybridization (FISH) and matrix-assisted laser-desorption-ionization time-of-flight mass-spectrometry (MALDI-TOF-MS) for the identification of *H. influenzae*, *H. parainfluenzae* and *H. haemolyticus* to species level.

**Methodology:**

A strain collection of 84 *Haemophilus* spp. comprising 50 *H. influenzae*, 25 *H. parainfluenzae*, 7 *H. haemolyticus*, and 2 *H. parahaemolyticus* including 77 clinical isolates was analyzed by FISH with newly designed DNA probes, and two different MALDI-TOF-MS systems (Bruker, Shimadzu) with and without prior formic acid extraction.

**Principal Findings:**

Among the 84 *Haemophilus* strains analyzed, FISH led to 71 correct results (85%), 13 uninterpretable results (15%), and no misidentifications. Shimadzu MALDI-TOF-MS resulted in 59 correct identifications (70%), 19 uninterpretable results (23%), and 6 misidentifications (7%), using colony material applied directly. Bruker MALDI-TOF-MS with prior formic acid extraction led to 74 correct results (88%), 4 uninterpretable results (5%) and 6 misidentifications (7%). The Bruker MALDI-TOF-MS misidentifications could be resolved by the addition of a suitable *H. haemolyticus* reference spectrum to the system's database. In conclusion, no analyzed diagnostic procedure was free of errors. Diagnostic results have to be interpreted carefully and alternative tests should be applied in case of ambiguous test results on isolates from seriously ill patients.

## Introduction

*Haemophilus influenzae*, a Gram-negative rod-shaped bacterium, is a major etiological agent of bacterial respiratory tract infections [1], [2], although the respiratory tract of 30% of healthy volunteers is asymptomatically colonized by this species [3]. *H. influenzae* is of particular importance for respiratory infections in children [4], while there is a decrease of pharyngeal *H. influenzae* colonization with increasing age [5]. *H. parainfluenzae*, in contrast, is a saprophyte that colonizes the upper respiratory tract and is frequently isolated from respiratory samples, but it hardly ever causes respiratory tract infections and only occasionally infectious endocarditis [6]. The etiological relevance of *H. haemolyticus* is controversial: Although it was previously considered as simply a commensal [7]–[9], recent data suggest occasional clinical relevance [10].

Fast and reliable discrimination of *H. influenzae* from the rarely relevant *H. parainfluenzae* and *H. haemolyticus* is important for therapeutic decisions and epidemiological assessments. *Haemophilus* spp. constitute about 10% of the culturable bacterial flora of the upper respiratory tract, with *H. influenzae* accounting for less than 2% of the *Haemophilus* burden in the pharynx [5], [11]. According to previous analyses, 10–40% of suspected *H. influenzae* isolates from non-sterile respiratory fluids such as sputum and nasopharyngeal secretions turned out to be commensal *H. haemolyticus* after ribosomal DNA sequence analysis, multilocus sequence analysis, DNA–DNA hybridization, and P6 gene sequencing [7], [12].

Classical biochemical identification of *H. influenzae* is usually achieved by various growth factor based-methods that make use of the fact that *H. influenzae* lacks the enzymatic capacity to convert delta-aminolevulinic acid (ALA) to protoporphyrin and therefore depends on factor X (heme) for growth. These tests take at least a couple of hours and are associated with considerable rates of erroneous identification of up to 10% [13], [14]. The frequently applied rapid biochemical identification kit API NH (bioMeriéux, Nürtingen, Germany) and the automatic differentiation tool VITEK 2 NH (bioMeriéux) produce false results in about 1–10% involving various *Haemophilus* and non-*Haemophilus* species [14]–[17]. Among several others, these misidentifications included identification of *H. parainfluenzae* as *H. influenzae* by API NH [15] as well as *H. influenzae* as *H. parainfluenzae* and *Haemophilus* spp., of *H. haemolyticus* as *H. influenzae*, and of *H. parainfluenzae* as *H. influenzae/haemolyticus* by VITEK 2 NH [17], stressing the difficulty of discrimination within the *Haemophilus* genus.

Conventional culture and biochemical differentiation are still the gold standard in many laboratories, although polymerase chain reaction (PCR) directly from sputum was shown to be superior to biochemical identification of *H. influenzae* in chronic obstructive pulmonary disease (COPD) patients [18]. Various (multiplex-)PCR protocols have been developed [18]–[21], but involve considerable effort. Thus, reliable but less complex identification methods are desirable.

Fluorescence *in situ* hybridization (FISH) is such an alternative molecular method for easy, rapid, and cost-effective identification of various pathogens [22]–[25]. It allows microscopic visualization of bacteria using fluorescence-labeled oligonucleotide probes, which bind to unique complementary target sites on ribosomal RNA. FISH has previously been applied for the identification of *H. influenzae* [22]. However, Hogardt's probe was only evaluated with a small number of organisms and showed homology with a few 16S rRNA sequences of non-target organisms submitted to GenBank (http://blast.ncbi.nlm.nih.gov) including *H. haemolyticus*. Therefore, it should be considered as a *H. influenza/H. haemolyticus* probe. To our knowledge, no probes for *H. parainfluenzae* and *H. haemolyticus* have yet been published, so we designed and evaluated new ones.

Another rapid technique for the identification of microorganisms, matrix-assisted laser desorption-ionization time-of-flight mass spectrometry (MALDI-TOF-MS), allows the identification of *Haemophilus* species within several minutes [26]. However, mass spectrometers are still expensive and accordingly not available in all diagnostic laboratories, particularly in resource-limited areas. Neither broad evaluation studies on MALDI-TOF-MS for the differentiation of *Haemophilus* spp. nor data on the identification of *H. haemolyticus* by MALDI-TOF-MS are available in the literature.

Here we describe the comparative evaluation of our newly designed FISH probes together with two different MALDI-TOF-MS platforms for the rapid discrimination of *H. influenzae*, *H. parainfluenzae*, and *H. haemolyticus* in a multi-center approach.

## Materials and Methods

### Strain Collection, Origin of Clinical Isolates and Coverage

A total of 94 strains were included in the analysis, comprising 50 *H. influenzae,* 25 *H. parainfluenzae,* 7 *H. haemolyticus,* 2 *H. parahaemolyticus,* 1 *Moraxella catarrhalis,* 1 *Stenotrophomonas maltophilia,* 1 *Acinetobacter baumannii,* 1 *Pseudomonas aeruginosa,* 1 *Klebsiella pneumoniae,* 1 *Streptococcus anginosus,* 1 *S. constellatus,* 1 *coagulase-negative Staphylococcus* sp., 1 *S. epidermidis,* and 1 *Micrococcus luteus.* The non-*Haemophilus spp.* were chosen as extended negative controls for the FISH evaluation because they are representatives of commensal or pathological flora of the naso-oro-pharyngeal mucous membranes that should not be confused with *Haemophilus* spp. in fluorescence-microscopic analysis.

The collection included 3 reference strains, i.e., *H. influenzae* ATCC 49, *H. haemolyticus* ATCC 33390, and *A. baumannii* ATCC 19606; 1 *H. influenzae* strain from German laboratory evaluations (“Ringversuche”); 3 well-characterized [27] *H. haemolyticus* strains 16N, 27p25, and PN24; and three strains from the strain collections of our Institutes, i.e., 1 *H. parahaemolyticus*, 1 *P. aeruginosa* and 1 *K. pneumoniae*. All of the other 84 strains were clinical isolates. The strains from the strain collections and all clinical isolates were provided by the Institutes for Microbiology, Virology and Hygiene of the University Hospitals Rostock and Ulm, Germany, during a 6-month collection period. In total, 60 clinical isolates were provided from Rostock (35 *H. influenzae*, 23 *H. parainfluenzae*, 1 *H. haemolyticus*, 1 *H. parahaemolyticus*) and 17 isolates from Ulm (13 *H. influenzae*, 2 *H. parainfluenzae*, 2 *H. haemolyticus*).

These 77 *Haemophilus* spp. were isolated from 26 sputa, 9 nasal swabs, 9 tracheal secretions, 7 broncho-alveolar lavages, 7 pharyngeal swabs (3 of them from known mucoviscidosis patients), 6 eye swabs, 5 nasal secretions, 2 bronchial secretions, 2 ear swabs, 1 palatine swab, 1 ossicle fragment, 1 maxillary sinus swab, and 1 peritonsillar abscess (Table 1) in routine microbiological diagnostic analyses. One isolate, a *H. parainfluenzae* strain, was isolated in the course of a recent anonymized study [28] at the University of Rostock. Patient-related data associated with the bacterial isolates, i.e., age and sex of the patients, are not presented for ethical considerations. No informed consent was obtained from the patients regarding the presentation of their personal data in this study. Accordingly, no further information regarding the origin of the strains can be shown.

**Table 1:** Distribution of *Haemophilus* spp. isolates by clinical material.

	*H. influenzae*	*H. parainfluenzae*	*H. haemolyticus*	*H. parahaemolyticus*	Total number of isolates
Distribution of clinical isolates					
Sputum	8 [31%]	17 [65%]	1 [4%]	-	26
Nasal swab	9 [100%]	-	-	-	9
Tracheal secretion	8 [89%]	1 [11%]	-	-	9
Broncho-alveolar lavage	4 [57%]	2 [29%]	1 [14%]	-	7
Pharyngeal swab	4 [57%]	1 [14%]	1 [14%]	1 [14%]	7
Eye swab	6 [100%]	-	-	-	6
Nasal secretion	5 [100%]	-	-	-	5
Bronchial secretion	1 [50%]	1 [50%]	-	-	2
Ear swab	2 [100%]	-	-	-	2
Palatine swab	-	1 [100%]	-	-	1
Ossicle fragment	-	1 [100%]	-	-	1
Maxillary sinus swab	1 [100%]	-	-	-	1
Peri-tonsillar abscess	-	1 [100%]	-	-	1
Total	48 [62%]	25 [32%]	3 [4%]	1 [1%]	77

The *Haemophilus* spp. isolates from Ulm were characterized phenotypically by Gram stain, colony morphology, cytochrome oxidase reaction, factors V (NAD) and X (heme) growth dependence on Mueller–Hinton agar, and the porphyrin test [29] prior to their inclusion in the study. These tests cannot distinguish *H. influenzae* from *H. haemolyticus*. The *Haemophilus* spp. isolates from Rostock were primarily identified by MALDI-TOF-MS with the Shimadzu system as described below. Among these, selected strains were further analyzed by API NH (n = 31) and the VITEK 2 NH (n = 8) cards (bioMérieux, Nürtingen, Germany) in Rostock because of operational prerequisites in the diagnostic algorithms (i.e., comparative testing of API NH and Shimadzu MALDI-TOF-MS during the implementation and harmonization of MALDI-TOF-MS technology in the diagnostic routine, technical reasons preventing MALDI-TO-MS analyses on the day of isolation, and in rare cases unexpected or uncertain MALDI-TOF-MS results). In these cases of biochemical differentiation, identification to species level was accepted at an identification score of ≥90%. All 84 *Haemophilus* spp. strains were analyzed by FISH and two MALDI-TOF-MS platforms in the scope of this study. All 7 non-*Haemophilus* isolates were identified by biochemical standard procedures, i.e., the respective API and VITEK 2 identification systems (bioMérieux).

### FISH Probe Design

Probes targeting *H. influenzae* (Hain 16S1251: 5′-TCG-CAG-CTT-CGC-TTC-CCT-3′; Hain 16S1253 5′-CGC-AGC-TTC-GCT-TCC-3′), *H. parainfluenzae* (Hapa 16S444: 5′-ACT-AAA-TGC-CTT-CCT-CGC-TAC-C-3′), and *H. haemolyticus* (Haha 16S1252: 5′-TCG-CAG-YTT-CGC-CAC-CCT-3′; Haha 16S1242 5′-TCG-CCA-CCC-TCT-GTA-TAC-G-3′) were designed using the ARB software (www.arb-home.de) [30], [31] and directly 5′-labeled with the red sulfoindocyanine dye Cy3 (Thermo Fisher Scientific, Ulm, Germany and Eurogentec Deutschland GmbH, Köln, Germany). The short *H. influenzae* probe Hain 16S1253 was used in conjunction with the non-labeled competitor probe Hain 16S1253comp 5′-CGC-AGC-TTC-GCC-ACC-3′ to prevent non-specific cross-binding to the closely related [7], [27] *H. haemolyticus* isolates. All probes were used in combination with a green-fluorescing carboxyfluorescein (FAM)-labeled eubacterial probe, EUB338, targeting nearly all bacteria [32].

### *In-silico* Evaluation of the FISH Probes

An *in-silico* evaluation of all newly designed probes was performed with the help of the software probeCheck (www.microbial-ecology.net/probecheck/) [33] as previously detailed by us [34], [35] using the sequence collection SILVA.

### Optimization of the Hybridization Conditions

All probes were designed to show optimal binding conditions at 46°C in hybridization buffer containing 30% formamide. Formamide concentrations in the hybridization buffer were increased in 5% steps and sodium chloride concentrations in the washing buffer were correspondingly reduced as described [36], [37] to optimize the binding stringency of the probes.

### FISH Procedure

**Figure 1:**
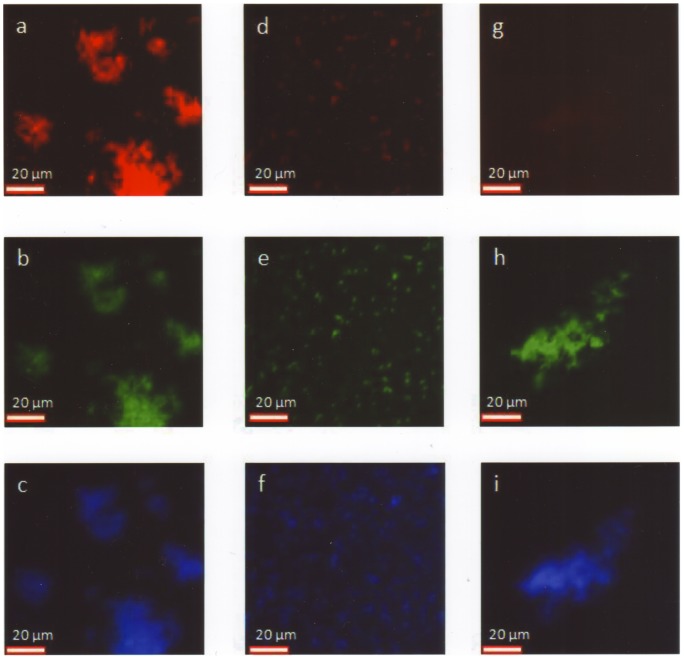
Perfect matches, borderline matches, and no matching by FISH. (a, d, g) Cy3-labeled species-specific probes. (b, e, h) FAM-labeled pan-eubacterial probe staining virtually all bacteria. (c, f, i) Non-intercalating DNA stain DAPI. (a–c) Perfect match of *H. haemolyticus* with a *H. haemolyticus* probe (Haha 16S1242). Fluorescence due to the species-specific probe is even higher than fluorescence due to the pan-eubacterial probe (intensity 5+). (d–f) Borderline match of *H. influenzae* with a *H. influenzae* probe/competitor probe combination (Hain 16S1253/Hain 16S1253comp). Fluorescence due to the species-specific probe is as high as fluorescence due to the pan-eubacterial probe for only few bacteria and is considerably lower for all other *H. influenzae* bacteria in a pure culture (intensity 3+). (g–i) No matching of *H. parahaemolyticus* with a probe with specificity for *H. influenzae*. Only slight autofluorescence is visible in the Cy3-channel (intensity 1+) compared with adequate fluorescence due to the pan-eubacterial probe.

FISH was performed at the Bernhard Nocht Institute for Tropical Medicine, Hamburg, Germany, as previously described [23], [36], [38]. In brief, all 84 *Haemophilus* spp. strains were grown on chocolate agar (BD, Franklin Lakes, NJ, USA), and the 10 non-*Haemophilus* spp. negative control strains on Columbia agar enriched with 5% sheep blood (BD). Colony material was diluted in 0.9% sodium chloride solutions to a density of MacFarland 4–5. Eight-well Diagnostica slides (Menzel-Gläser, Braunschweig, Germany) with smears of these solutions were air-dried and fixed in 100% methanol for 10 minutes. Only one strain per slide was applied. Hybridization was performed for 1 hour at 46°C in hybridization buffer at the optimized formamide concentration. The slides were then rinsed with pre-heated washing buffer [36] and afterward washed for an additional 15 minutes in washing buffer at 46°C [36]. Subsequently, they were covered with “Vectashield with DAPI” (Vector Laboratories, Burlingame, CA, USA), a mounting medium containing the non-intercalating DNA stain 4′,6-diamidino-2-phenylindole (DAPI) as a counterstain to demonstrate the presence of bacterial DNA. Microscopic examination was performed with an upright Leica.DM5000B fluorescence microscope (Leica, Wetzlar, Germany) equipped with a Leica DFC 360 FX camera at 630× magnification. Images were acquired and processed using the Openlab 5.1 software (Improvision, Coventry, UK). Evaluation of fluorescence intensity was based upon subjective assessment from 0 to 5 by experienced investigators without automated measurement. Definitive species identification by sequence analysis was not available at this time. Fluorescence intensity of ≤2 was considered as negative, fluorescence intensity of ≥4 as positive; fluorescence intensity of 3 was considered as borderline reaction (Figure 1).

For a FISH-based diagnosis to species level, all results of all respective species-specific probes had to be positive, and results of all probes with specificity for other *Haemophilus* spp. had to be negative.

### Bruker MALDI-TOF-MS

MALDI-TOF-MS analyses using a Bruker Daltonics Microflex LT mass spectrometer operated by the MALDI biotyper operation control (Billerica, MA, USA) and the MALDI-Biotyper 2.1 software were performed for all 84 *Haemophilus* strains at the Institute for Microbiology, Virology and Hygiene, University Hospital Eppendorf, Hamburg, Germany. As recommended by the manufacturer, all samples were prepared for mass spectrometry measurement by formic acid extraction [39], [40]. Colony material was suspended in 300 µL distilled water, mixed with 900 µL ethanol, and repeatedly centrifuged at 13,000 *g* for 2 minutes to discard the supernatant and remove residual ethanol. The resulting pellet was re-suspended in 35 µL 70% formic acid and the same volume of acetonitrile, incubated at room temperature for 5 minutes, and centrifuged at 13,000 *g* for 2 minutes. Volumes of supernatant of 1 µL were air-dried in triplicate on 96-well ground-steel targets (Bruker Daltonics), overlaid with 1.5 µL matrix solution (saturated alpha-cyano-4-hydroxycinnamic acid in 50% acetonitrile with 2.5% trifluoroacetic acid), air-dried again at room temperature, and analyzed by mass spectrometry with 280 shots per sample spot using the recommended instrument settings for bacterial identifications. In addition, all clinical isolates were also investigated by the direct sample deposition protocol that is preferentially applied under routine conditions as an alternative to the more time-consuming extraction procedure. To this end, single colonies were spotted in duplicate on 96-well ground-steel targets, overlaid with 1.5 µL matrix solution and air dried at room temperature.

Automated identification was performed with the MALDI-Biotyper 2.1 software, database version 3.2.1.1 (Bruker Daltonics), including 4110 spectra. Species-level identification was assumed if the logarithmic identification score, expressing the degree of concordance with the best matching spectrum from the reference database, reached or exceeded the manufacturer's proposed threshold of 2.0. Identification scores between 1.7 and <2.0 were considered a genus identification.

The results with and without prior formic acid extraction were compared with Student's unpaired t-test with unequal variances using Microsoft Excel 2007®.

### Shimadzu MALDI-TOF-MS

All *Haemophilus* spp. strains were analyzed after 24 hours of growth with a Shimadzu/Kratos “AXIMA Assurance” MALDI-TOF mass spectrometer (SHIMADZU Deutschland GmbH, Duisburg, Germany) at the Institute for Microbiology, Virology and Hygiene, University Hospital of Rostock, Rostock, Germany under routine conditions according to the manufacturer's instructions. In brief, single colonies were picked with inoculation loops and the colony material was inoculated on 48-well ground-steel targets. Subsequently, 1 µL alpha-cyano matrix (“ripac Labor”, Potsdam Gollen, Germany) was added, mixed with the colony material using a pipette tip, and air-dried at room temperature. Automated identification was performed via the ID Professional AgnosTec SARAMIS database version 4.07, system version 3.4.1.10, including 45,894 spectra and 3,382 super spectra deduced from these spectra. Identifications to species level were accepted if the automated comparison with deposited reference spectra led to ≥80% identity as defined by the deposited super spectra, recommended by the manufacturer and confirmed by a previous in-house validation in Rostock (data not shown). Results between 70% and <80% were considered unacceptable; lower identity scores were not provided by the software but were documented as “not identified.” However, in some instances of identification scores <80%, the software at least provided “Hints” for the possibility of the presence of the indicated organism. In case of failed identification or unacceptable low identity scores, the laboratory's diagnostic algorithms directed the use of alternative diagnostic procedures.

### Diagnostic Gold Standard in Case of Conflicting Results

Conflicting results of biochemical procedures, FISH and MALDI-TOF-MS were resolved by 16S rRNA gene sequencing with the primers 5′-AGA-GTT-TGA-TCM-TGG-CTC-AG-3′ and 5′-CCG-TCA-ATT-CMT-TTR-AGT-TT-3′ (spanning the base positions 1–917 of the 16S rRNA gene, NCBI accession number NC_009085.1) as described by Cilia et al. [41]. The 917-base sequence was matched via NCBI BLAST (www.blast.ncbi.nlm.nih.gov) with deposited bacterial sequences. The closest match established the final diagnosis, and the final concordance numbers were based on these “resolved” species identities. The interpretation of sequencing results was based on the CLSI (Clinical and Laboratory Standards Institute) guideline MM18-A “Interpretive Criteria for Identification of Bacteria and Fungi by DNA Target Sequencing; Approved Guideline” [42] as described in previous studies [43]. In detail, ≥99% identity was demanded for species identification as suggested for glucose non-fermenting Gram-negative bacilli. In case of poorer results, PCR and sequencing were repeated. As cases of confusion of *H. haemolyticus* with *H. influenzae* and vice versa are known from previous studies [7], [27], which would potentially have resulted in incorrect database entries, exceptions from the generally applied rule of 0.8% separation between different species were accepted for these two species, but strictly if only single database-entries of the one species were found among a vast majority of the other, within the ≥99% identity range. Confirmation testing based on 23S rRNA or ribosomal protein B (*rpoB*) gene sequencing was not performed.

### Ethics

The study reported here required neither ethical approval nor informed consent, because it did not involve human participants. No patient-related data were analyzed. No primary human sample materials were used, only bacterial isolates from routine diagnostic procedures or strain collections. All analyzed clinical strains were acquired during routine diagnostic procedures. Accordingly, no acquisition of patient samples for the study was undertaken.

## Results

### Fluorescence *In Situ* Hybridization (FISH)

#### *In-silico* evaluation and identification of optimal hybridization conditions

All newly designed species-specific probes were shown *in silico* to have sequence homology with a few single bacterial 16S rRNA gene sequences of clinically relevant non-target bacteria, including closely related *Haemophilus* species within the two-base-pair-mismatch range, in which cross-bindings might occur. In addition to these *Haemophilus* species, sequence homologies were observed with uncultured or environmental bacteria; Gram-positive bacteria such as *Actinobacillus* spp., *Bacillus* spp., *Erysipelothrix rhusiopathiae*, *Lactobacillus* spp., and *Listeria monocytogenes*; anaerobic bacteria such as *Clostridium* spp.; or single sequences of Gram-negative, rod-shaped bacteria such as *Legionella* spp., *Shewanella* spp., and non-pathogenic *Aggregatibacter* spp. Several of these homologies were shared by both probes with intended specificity for one *Haemophilus* species. However, colonies of the respective non-*Haemophilus* spp. are easy to distinguish from *Haemophilus* spp. on the agar plate, reducing their impact on the interpretation of FISH results to negligible.

The design of a competitor probe for the short *H. influenzae* probe Hain 16S1253 was necessary due to close sequence homology with *H. haemolyticus* [27] to reduce the probability of non-specific cross-binding. The close sequence homology of *H. influenza* and *H. haemolyticus* [27] prompted the design and use of two probes with specificity for *H. haemolyticus*. The combined use of two different probes for this species was intended to falsify potential non-specific binding of one probe by the lack of binding of the other. Such contradictory results, e.g., the binding of only one out of two *H. influenzae* specific probes, have to be interpreted as uninterpretable results but not as misidentifications.

The design of absolutely specific probes by *in-silico* evaluation was virtually impossible because of the close genetic homology of the 16S RNA gene within the *Haemophilus* genus [7], [27]. In detail, the *H. influenzae* probes Hain 16S1251 and Hain 16S1253 showed identical *in-silico* cross-matching with one *H. haemolyticus* sequence each, and the *H. haemolyticus* probe Haha 16S1242 with as many as three *H. influenzae* sequences in a probeCheck (www.microbial-ecology.net/probecheck/) [33] analysis using the SILVA sequence collection. These identical cross-matches in SILVA were identified within 10,000 matches for the two *H. influenzae* probes, 152 matches for the *H. parainfluenzae* probe, as well as 2,422 and 4,217 matches for the *H. haemolyticus* probes Hhaem1242 and Hhaem1252, respectively, within the two-base-pair-mismatch range. However, the reliability of sequence databases is compromised by potential misidentifications due to the above-mentioned diagnostic pitfalls, so a thorough *in-vitro* evaluation was unavoidable.

Optimization of binding stringency of the newly designed FISH probes by increasing the formamide concentration in the hybridization buffer and correspondingly reducing the sodium chloride concentration in the washing buffer confirmed optimal binding of the probes under the standard conditions of 46°C and 30% formamide in the hybridization buffer. These conditions allowed for the combined use of all probes on a multiple-well slide.

#### *In-vitro* evaluation of the FISH probes

**Figure 2:**
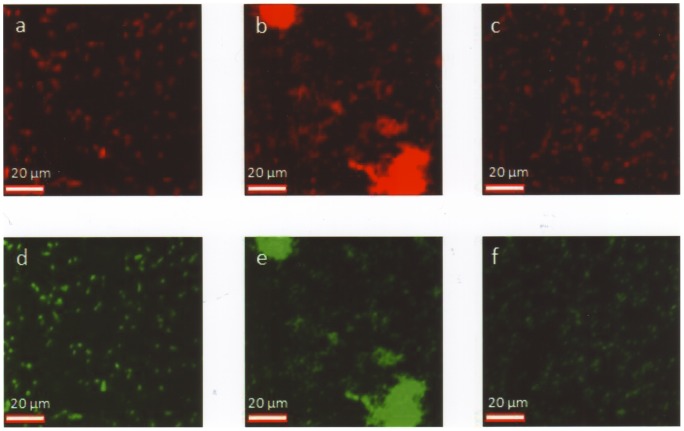
*Haemophilus* spp. identified by FISH. (a, d) Smooth layers of *H. influenzae*, corresponding positions. (b, e) Clusters of *H. parainfluenzae* (prominent in the white circle), corresponding positions. (c, f) Smooth layers of *H. haemolyticus*, corresponding positions. (a–c) Cy3-labeled species-specific probes. (d–f) FAM-labeled pan-eubacterial probe staining virtually all bacteria.

The collection of 84 clinical isolates and 10 well-defined reference strains described above was investigated by FISH (Figure 2, Table 2).

**Table 2:** Evaluation of the newly designed *Haemophilus* probes by FISH.

Probe/competitor probe	Designed to identify	Reaction with				
		*H. influenzae*	*H. parainfluenzae*	*H. haemolyticus*	*H. parahaemolyticus*	Others*
Hain 16S1251	*H. influenzae*	50/50	0/25	**1/7**	**1/2**	0/10
Hain 16S1253/Hain 16S1253comp	*H. influenzae*	47/50†	0/25	0/7	0/2	0/10
Hapa 16S444	*H. parainfluenzae*	0/50	25/25	0/7	0/2	0/10
Haha 16S1252	*H. haemolyticus*	**1/50**	**2/25**	7/7	0/7	0/10
Haha 16S1242	*H. haemolyticus*	**3/50**	**2/25**	7/7	0/7	0/10

* Non-*Haemophilus* negative control strains including isolates from the commensal or pathological flora of the upper respiratory tract including *Moraxella catarrhalis*, *Stenotrophomonas maltophilia*, *Acinetobacter baumannii*, *Pseudomonas aeruginosa*, *Klebsiella pneumoniae*, *Streptococcus anginosus*, *S. constellatus*, coagulase-negative *Staphylococcus* spp., *S. epidermidis*, and *Micrococcus luteus*. **Bold type** indicates incorrect binding. †Three out of 50 strains were missed.

The *H. influenzae* probe Hain 16S1251 correctly identified all 50 *H. influenzae* strains and excluded all 25 *H. parainfluenzae* strains and all 10 non-*Haemophilus* spp. strains. However, we reproducibly observed a cross-binding with 1 out of 2 *H. parahaemolyticus* strains and a borderline cross-binding with 1 out of 7 *H. haemolyticus* strains.

Therefore, the shorter *H. influenzae probe* Hain 16S1253 was designed and used in combination with the competitor probe Hain 16S1253comp. This probe/competitor probe combination did not show any non-specific cross-binding. However, it reproducibly missed 3 out of 50 *H. influenzae* strains. Omitting the competitor probe also failed to generate a fluorescence signal with the missed strains.

The *H. parainfluenzae* probe Hapa 16S444 correctly identified all 25 *H. parainfluenzae* strains and excluded all 69 non-target strains (Table 2).

The *H. haemolyticus* probe Haha 16S1252 correctly identified all 7 H. haemolyticus strains. However, there were three reproducible borderline cross-reactions with one *H. influenzae* strain and two *H. parainfluenzae* strains (Table 2) among the 87 non-target strains.

The *H. haemolyticus* probe Haha 16S1242 correctly identified all 7 *H. haemolyticus* strains as well. There were four reproducible borderline cross-reactions with two *H. influenzae* strains and two *H. parainfluenzae* strains, respectively, and one reproducible cross-binding to one *H. influenzae* strain (Table 2) among the 87 non-target strains.

The non-specific, partially borderline cross-bindings of the *H. haemolyticus* probes Haha 16S1252 and Haha 16S1242 comprised different strains. No non-*H. haemolyticus* strain showed non-specific binding with both *H. haemolyticus* probes (Table 2).

Including all 5 used *Haemophilus* probes, there were 3 missed bindings (0.6%), 2 cross-bindings (0.4%) and 8 borderline cross-bindings (1.7%) in the 470 FISH reactions evaluated. All of these 2.8% incorrect results affected different strains. Consequently, the concerted use of all *Haemophilus*-specific probes on multi-well slides led to correct identifications of 43 out of 50 *H. influenzae* strains (86%), 21 out of 25 *H. parainfluenzae* strains (84%), and 6 out of 7 *H. haemolyticus* strains (86%). Further, the procedure excluded all tested non-*Haemophilus* strains (100%) and 1 out of 2 *H. parahaemolyticus* strains (50%) (Table 3).

**Table 3:** Identification of *Haemophilus* spp. by FISH and two MALDI-TOF-MS procedures.

Analyzed strains	*H. influenzae*	*H. parainfluenzae*	*H. haemolyticus*	*H. parahaemolyticus*	Others*
Diagnostic procedure	Multiple-probe FISH				
Correctly identified	43/50	21/25	6/7	1/2	0/10
Failed identifications	7	4	1	1	-
**Misidentifications**	**-**	**-**	**-**	**-**	**-**
Diagnostic procedure	Bruker MALDI-TOF-MS with prior formic acid extraction				
Correctly identified	50/50	22/25	0/7†	2/2	-
Failed identifications	-	3	1	-	-
**Misidentifications**	**-**	**-**	**6**	**-**	**-**
Diagnostic procedure	Bruker MALDI-TOF-MS without prior formic acid extraction				
Correctly identified	49/50	18/25	0/3†	1/2	-
Failed identifications	1	7	-	1	-
**Misidentifications**	**-**	**-**	**3**	**-**	**-**
Diagnostic procedure	Shimadzu MALDI-TOF-MS without prior formic acid extraction				
Correctly identified	41/50	17/25	0/7	1/2	-
Failed identifications	8	6	5	-	-
**Misidentifications**	**1**	**2**	**2**	**1**	**-**

* Non-*Haemophilus* negative control strains including isolates from the commensal or pathological flora of the upper respiratory tract including *Moraxella catarrhalis*, *Stenotrophomonas maltophilia*, *Acinetobacter baumannii*, *Pseudomonas aeruginosa*, *Klebsiella pneumoniae*, *Streptococcus anginosus*, *S. constellatus*, coagulase-negative *Staphylococcus* spp., *S. epidermidis*, and *Micrococcus luteus*.

† Correct identification was possible after implementation of a newly established *H. haemolyticus* spectrum based on repeated measurements of the reference strain *H. haemolyticus* ATCC 33390.

**Bold type** indicates misidentifications to species level.

The combined use of all newly designed FISH probes did not lead to any misidentifications but only to failed identifications due to uninterpretable results with the minority of isolates that would have led to identification by other diagnostic procedures (Table 3). In total, failed identifications were detected in 13 out of 94 (13.8%) identifications or 13 out of 84 (15.5%) if only the *Haemophilus* spp. are considered.

### Bruker MALDI-TOF-MS

Bruker MALDI-TOF-MS analysis after formic acid extraction correctly identified 50 out of 50 *H. influenzae* strains (100%). The mean identification score was 2.30 (±0.08) with and 2.27 (±0.12) without prior formic acid extraction. The difference was not significant (p = 0.14). A score >2.0 was achieved in all instances after formic acid extraction and for 49 out 50 strains (98%) without the extraction procedure, while it was just missed for one strain (2%) with a best identification score of 1.99.

**Figure 3:**
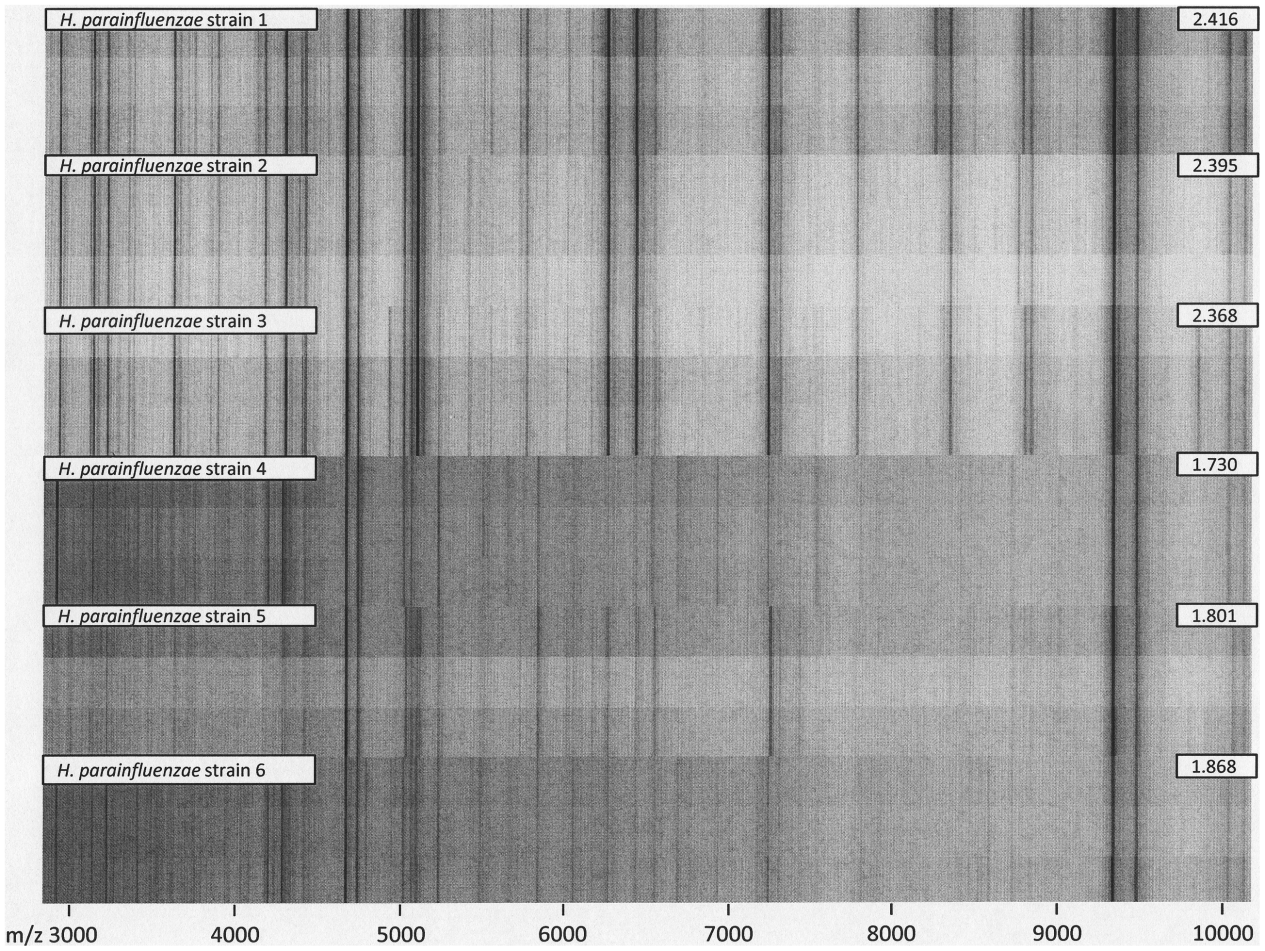
Formic acid extraction triplicate spectra from the six *H. parainfluenzae* study isolates with the highest (top) and lowest (bottom) MALDI Biotyper identification scores. Inscribed numbers denote the highest score from triplicate measurements. Scores ≥2.0 constitute species-level identification. Lower-scoring spectra show reduced signal intensity and signal-to-noise ratio, resulting in reduced peak detection rates. For non-*H. haemolyticus* study isolates, failed species-level identification with the Bruker mass spectrometry fingerprinting system could be attributed to suboptimal spectrum quality rather than insufficient database coverage.

The procedure led to best matches with the *H. parainfluenzae* spectrum for 25 out of 25 *H. parainfluenzae* isolates (100%). The mean identification score was 2.17 (±0.18) with and 2.13 (±0.20) without prior formic acid extraction. The difference was not significant (p = 0.48). The manufacturer-recommended score of >2.0 for identification to species level was achieved for 22 out of 25 strains (88%) after the extraction procedure. The other three strains led to best scores of 1.73, 1.80 and 1.86, respectively, in spite of repeated testing, not allowing formal identification to species level. Without formic acid extraction, 18 out of 25 strains (72%) yielded identification scores >2.0, while the scores for the other 7 *H. parainfluenzae* strains were 1.63, 1.76, 1.82, 1.94, 1.96, and twice 1.97, respectively. Such lower-scoring spectra showed reduced signal intensity and signal-to-noise ratio, resulting in reduced peak detection rates. Accordingly, failed species-level identification with the Bruker mass spectrometry fingerprinting system could be attributed to suboptimal spectrum quality rather than insufficient database coverage (Figure 3).

Seven out of 7 *H. haemolyticus* strains (100%), including the well-characterized strains ATCC 33390, 16N, 27p25, and PN24, were misidentified as *H. influenzae* using the MALDI-Biotyper 2.0 database. The respective best identification scores for *H. influenzae* after formic acid extraction were 1.92, 2.05, 2.08, 2.14, 2.15, 2.25 and 2.27, respectively, resulting in 1 failed species identification and 6 incorrect species identifications. The three clinical *H. haemolyticus* isolates were also directly applied to MALDI-TOF-MS, leading to identification scores for *H. influenzae* of 2.03, 2.06 and 2.22, respectively, resulting in incorrect species identifications. The mean identification score for the mismatching with *H. influenzae* was 2.12 (±0.12) with and 2.10 (±0.10) without prior formic acid extraction. The difference was not significant (p = 0.78).

As no *H. haemolyticus* reference spectrum had been provided with the version of the Bruker database that was employed, an in-house *H. haemolyticus* reference spectrum was compiled according to the manufacturer's instructions from repeated measurements of the *H. haemolyticus* reference strain ATCC 33390, which allowed for correct identification of all seven *H. haemolyticus* strains. Based on this new reference spectrum, the identification scores were 2.16, 2.22, 2.12, 2.24, 2.39, 2.41 and 2.67 (for ATCC 33390 itself), respectively, after formic acid extraction. Best identification scores of 2.04, 2.18 and 2.37 were achieved without the extraction procedure for the three clinical *H. haemolyticus* isolates. The mean identification score values were 2.31 (±0.19) with and 2.20 (±0.16) without prior formic acid extraction. The difference was not significant (p = 0.38).

**Figure 4:**
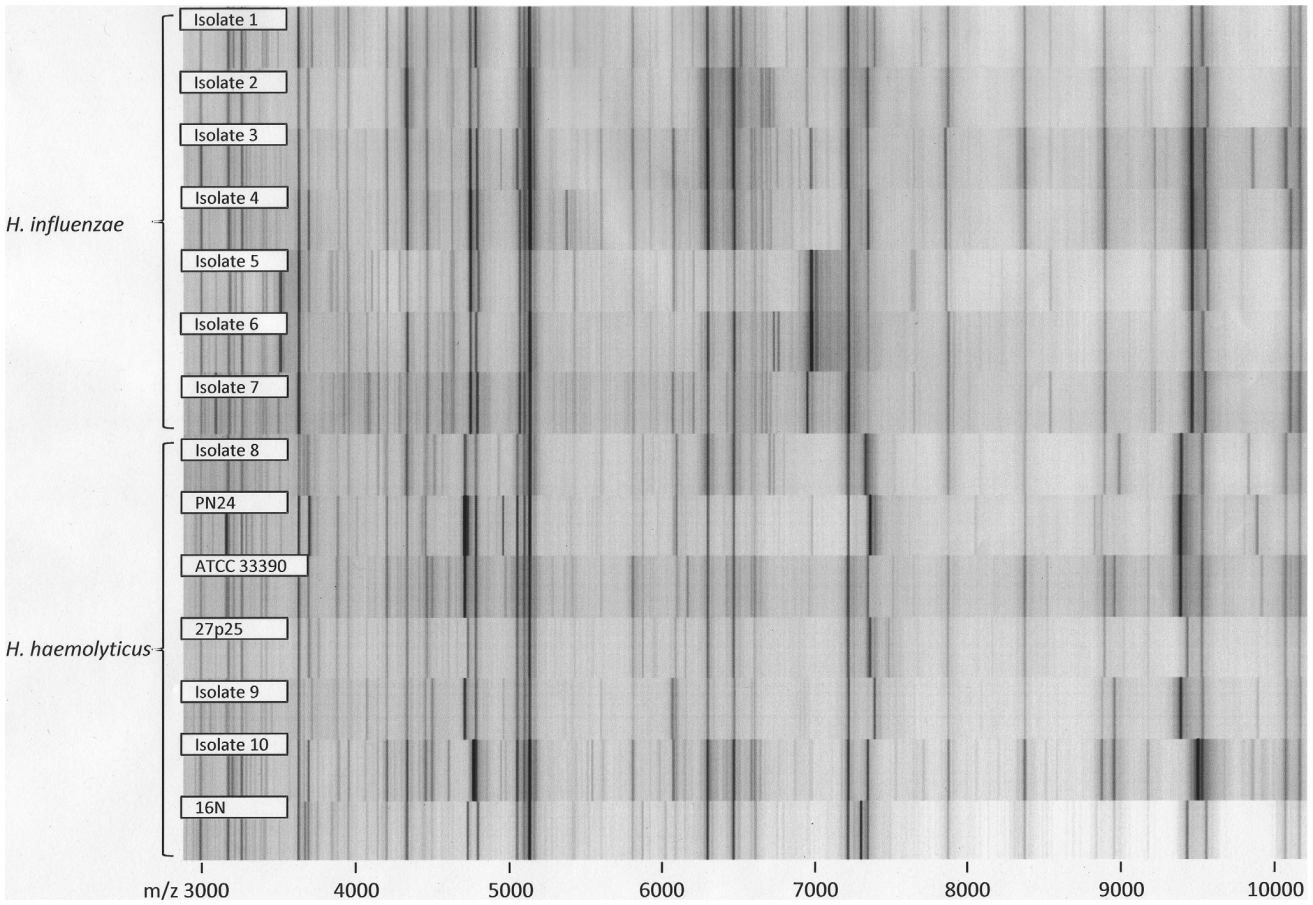
Opposed spectra of 7 *H. influenzae* and 7 *H. haemolyticus* spectra in gel view. The comparison illustrates high overall spectral similarity between the species, with considerable intraspecies variability.

**Figure 5:**
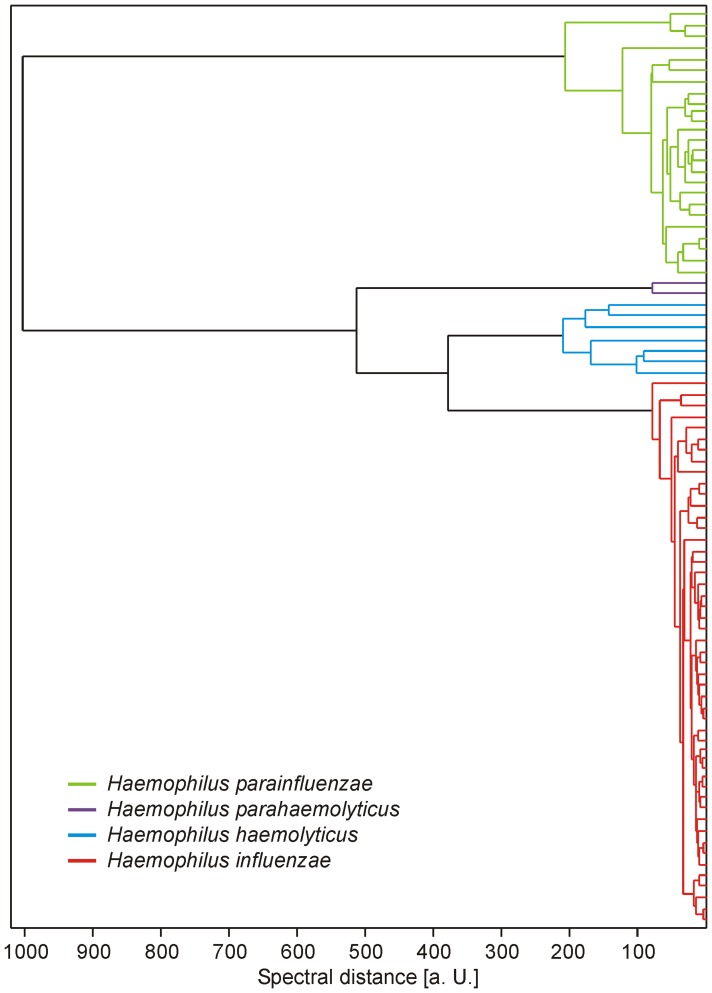
Spectral similarity between consensus spectra (MSPs) from formic acid extraction triplicate spectra of study isolates. The Biotyper 2.1 software with default parameter settings has been used for MSP creation and dendrogram calculation. Spectra of the four investigated *Haemophilus* species formed distinct clusters. Closest similarity was observed between *H. influenzae* and *H. haemolyticus* isolates. (a. U.  =  arbitrary units).

Direct comparison of the measured *H. haemolyticus* and *H. influenzae* spectra illustrated high overall spectral similarity between both species with considerable intraspecies variability (Figure 4). To provide an example, the spectral distance of the identification scores to the next match with a *H. influenzae* spectrum was >0.2 for no more than 3 out of 7 *H. haemolyticus* strains; for the other 4 it was even closer. During cross-testing of the new *H. haemolyticus* spectrum with the spectra of all other *Haemophilus* spp. strains that were analyzed in this study, no misidentification was observed. In detail, the new *H. haemolyticus* spectrum was never the best match for any of the analyzed non-*H. haemolyticus* strains. Six out of 50 *H. influenzae* strains (12%) matched with an identification score of >2.0 with the new *H. haemolyticus* spectrum, but these all matched with higher scores to at least one of the *H. influenzae* reference spectra, so no misidentifications occurred. The high overall spectral similarity of the measured *H. haemolyticus* and *H. influenzae* spectra is illustrated in Figure 5, although clusters are still distinguishable.

Two *H. parahaemolyticus* strains were correctly identified after formic acid extraction. Both scores were >2.0, the mean value being 2.16 (±0.02). Without the extraction procedure, one strain was missed with a score of 1.74, the mean value of both scores being 1.94 (±0.29). The difference of the mean values was not significant (p = 0.49).

Thus the MALDI-TOF-MS procedure led to 100% correct identifications of *H. influenzae* (50/50) and *H. parahaemolyticus* (2/2), 88% correct identifications of *H. parainfluenzae* (22/25), and 0% of *H. haemolyticus* (0/7). Identification of the *H. haemolyticus* strains required the establishment of a new reference spectrum. Altogether, in 84 identifications, there were 6 misidentifications (7.1%) and 4 failed identifications to species level (4.8%) (Table 3).

**Figure 6:**
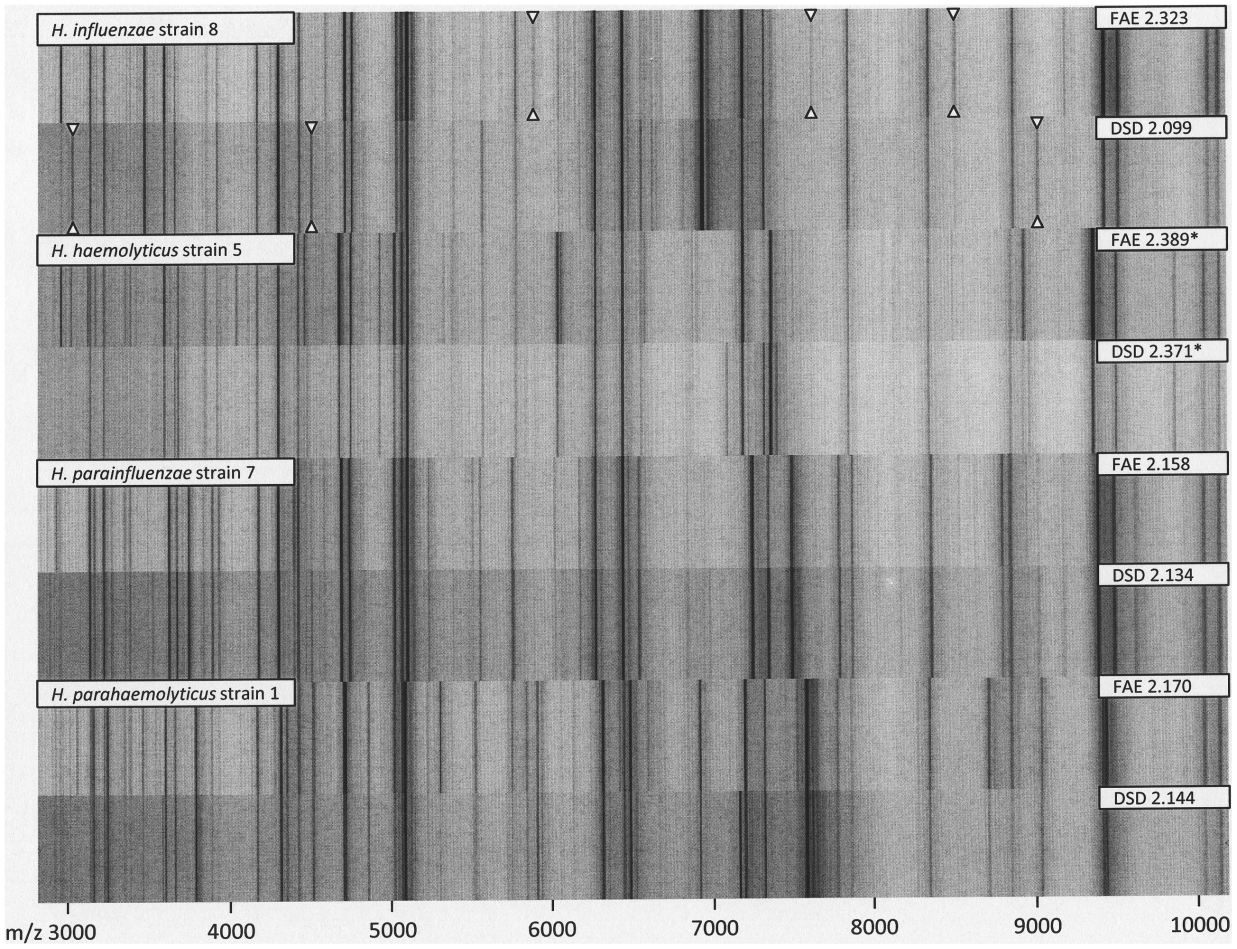
A comparison between formic acid extraction (FAE) and direct sample deposition (DSD) spectra for selected study isolates. Inscribed numbers denote the Biotyper identification score. Scores for *H. haemolyticus* (marked with an asterisk) have been obtained from comparison with an in-house reference spectrum. Although reproducible spectral differences could be observed for some isolates (highlighted with triangles in the topmost spectrum pair), mean identification scores for both techniques did not differ significantly.

Although reproducible spectral differences were observed in direct comparison between formic acid extraction and direct sample deposition spectra for selected study isolates, mean identification scores for the two techniques did not differ significantly (Figure 6).

### Shimadzu MALDI-TOF-MS

Shimadzu MALDI-TOF-MS analysis without prior in-house extraction under essentially routine conditions – i.e., one try with MALDI-TOF-MS and alternative diagnostic procedures in case of failed identification or unacceptably low identity scores by MALDI-TOF-MS – led to the correct identification of 41 out of 50 *H. influenzae* strains (82%). The mean identification score for these correctly identified strains was 97.0% (±5.1%). All correctly identified strains showed a score >80%. One out of 50 *H. influenzae* strains (2%) was misidentified as *H. parainfluenzae* with a score of 92.7%. Identification failed for 8 out of 50 strains (16%) due to poor matches with deposited reference spectra, as the identification scores were too low to match with any deposited spectrum, even to genus level.

Seventeen out of 25 *H. parainfluenzae* strains (68%) were correctly identified. The mean identification score for the correctly identified strains was 93.9% (±5.7%). The scores for all respective strains were above the 80% cut-off for acceptable identifications. Two out of 25 strains (8%) were misidentified as *H. influenzae*, with identification scores of 94% and 82%, respectively. An additional 2 out of 25 strains (8%) showed poor matching to deposited reference spectra with 77.6% identification score each for *Haemophilus* spp. in one instance and in the other instance the software indicated the possibility of *H. parainfluenzae*. Identification failed completely for 4 out of 25 *H. parainfluenzae* strains (16%) due to poorly matching results.

Among 7 *H. haemolyticus* strains, the software indicated the possibility of *H. haemolyticus* in three instances (42.9%). However, the associated poor identification scores (<70%) are regarded as unacceptable by the manufacturer. In the other four instances, *H. haemophilus* was misidentified as *H. influenzae*. In two out of these four instances (28.6%), the scores were acceptable at 86.5% and 93.7%, respectively, resulting in misidentifications. In the other two instances (28.6%), the scores were unacceptably low at 78.1% and 79.3%, respectively.

One out of 2 *H. parahaemolyticus* strains was correctly identified with a score value of 84%. The other strain was misidentified as *H. influenzae* with a score of 99.9%.

In total, there were 6 (7.1%) misidentifications and 19 (22.6%) failed identifications in 84 analyses of *Haemophilus* spp. by Shimadzu MALDI-TOF-MS (Table 3).

### Biochemical Identification

In the diagnostic workflow of the clinical isolates, several *Haemophilus* spp. were also analyzed by biochemical differentiation systems. In total, 31 included strains were analyzed by API NH in Rostock. Among 19 analyzed *H. influenzae* strains, 17 (89.5%) were correctly identified as *H. influenzae* by API NH with a mean identification score of 99.6% (±1.2%). All scores were ≥95%. Two *H. influenzae* strains (10.5%) were misidentified as *H. parainfluenzae* with identification scores of 98.7% and 99.9%, respectively.

Among 12 *H. parainfluenzae* strains, 9 (75%) were technically correctly identified by API NH with a mean identification score of 94.4% (±19.1%). Eight of these 9 scores were >99%; the ninth score was 42.2%, which was below the identification threshold and therefore an incorrect result. API NH indicated only the possibility of *H. parainfluenzae* in a further instance (8.3%), but matching was so poor that no identity score was printed. Two out of 12 *H. parainfluenzae* strains (16.7%) were misidentified as *H. influenzae* with identification scores of 91% and 97.1%, respectively.

In total, among 31 API NH analyses, there were 4 misidentifications (12.9%) and 2 failed identifications (6.5%).

Eight strains were analyzed by VITEK 2 NH-cards in Rostock. VITEK 2 correctly identified 4 out of 4 *H. influenzae* strains with a mean identification score of 96.3% (±2.2%). No score was <90%. One of these strains was also identified by API NH with a score of 99.9%. Three *H. parainfluenzae* strains were technically correctly identified by name with a mean identification score of 77.7% (±25.0%). The scores of 2 out of 3 strains (66.7%) were <90%, resulting in incorrect results due to failed identification to species level because of scores below the identification threshold. One of these two strains for which the VITEK 2 NH identification score was 49.23% showed an API NH identification score of 42.4% for *H. parainfluenzae*. One *H. haemolyticus* strain was misidentified as *H. parainfluenzae*, but identification to species level had to be rejected, because the identification score of 85% was below the acceptance level. In total, among 8 VITEK 2 NH analyses, there were 3 failed identifications to species level (37.5%).

All 17 *Haemophilus* spp. strains from Ulm, including 13 *H. influenzae* strains, 2 *H. parainfluenzae* strains, and 2 *H. haemolyticus strains*, were identified by cytochrome oxidase reaction, factors V (NAD) and X (heme) growth dependence, and the porphyrin test. While 100% of *H. influenzae* and *H. parainfluenzae* were correctly identified, both *H. haemolyticus* strains were misidentified as *H. influenzae* by the traditional biochemical approaches, as expected. Thus, among 17 analyses, there were two misidentifications (11.8%).

### Conflicting Diagnostic Results

Altogether, conflicting diagnostic results comprising failed identifications and misidentifications to species level as described above that required the use of 16S rRNA gene sequencing for the identification of the respective *Haemophilus* spp. to species level were observed for 29 out of 77 clinical isolates (38%) and one *H. parahaemolyticus* strain from a strain collection (Table 4, readable sequence fragments in Table S1). The well-characterized *H. haemolyticus* strains [27] that were donated from the University of Aarhus were not sequenced by us.

**Table 4:** Combined presentation of misleading or contradictory patterns of diagnostic results from 29 out of 77 clinical *Haemophilus* spp. isolates, 4 well-characterized *H. haemophilus* strains (donation from the University of Aarhus) and 1 *Haemophilus parahaemolyticus* strain from a strain collection.

Species identity (as defined by 16S rDNA gene analysis)	At least borderline binding of FISH probes with specificity for	Bruker MALDI-TOF- MS result (best match)	Shimadzu MALDI- TOF-MS result (best match)	API NH result (best match)	VITIK II NH result (best match)	Number of instances
*H. influenzae*	*H. influenzae* **(one out of two probes)**	*H. influenzae*	*H. influenzae*	-	-	1
	*H. influenzae* **(one out of two probes)**	*H. influenzae*	*H. influenzae*	*H. influenzae*	-	1
	*H. influenzae* **(one out of two probes)**	*H. influenzae*	**No I.D.**	*H. influenzae*	-	1
	*H. influenzae* (both probes), **H. haemolyticus (one out of two probes)**	*H. influenzae*	*H. influenzae*	-	-	2
	*H. influenzae* (both probes), ***H. haemolyticus*** ** (one out of two probes)**	*H. influenzae*	*H. influenzae*	-	*H. influenzae*	1
	*H. influenzae* (both probes), **H. haemolyticus (one out of two probes)**	*H. influenzae*	**No I.D.**	*H. influenzae*	-	1
	*H. influenzae* (both probes)	*H. influenzae*	**No I.D.**	*H. influenzae*	-	4
	*H. influenzae* (both probes)	*H. influenzae*	***H. parainfluenzae***	-	-	1
	*H. influenzae* (both probes)	*H. influenzae*	**No I.D.**	***H. parainfluenzae***	-	2
*H. parainfluenzae*	*H. parainfluenzae*, ***H. haemolyticus*** ** (one out of two probes)**	*H. parainfluenzae*	*H. parainfluenzae*	-	-	2
	*H. parainfluenzae*, ***H. haemolyticus*** ** (one out of two probes)**	*H. parainfluenzae*	*H. parainfluenzae*	*H. parainfluenzae*	-	1
	*H. parainfluenzae*, ***H. haemolyticus*** ** (one out of two probes)**	*H. parainfluenzae*	*H. parainfluenzae*	-	*H. parainfluenzae*	1
	*H. parainfluenzae*	*H. parainfluenzae*	***H. influenzae***	-	-	1
	*H. parainfluenzae*	*H. parainfluenzae*	***H. influenzae***	***H. influenzae***	-	1
	*H. parainfluenzae*	*H. parainfluenzae*	***Haemophilus*** ** spp.**	*H. parainfluenzae*	-	1
	*H. parainfluenzae*	*H. parainfluenzae*	***Haemophilus*** ** spp.**/*H. parainfluenzae*	-	*H. parainfluenzae*	1
	*H. parainfluenzae*	*H. parainfluenzae*	**No I.D.**	-	-	1
	*H. parainfluenzae*	*H. parainfluenzae*	**No I.D.**	*H. parainfluenzae*	-	1
	*H. parainfluenzae*	*H. parainfluenzae*	**No I.D.**	*H. parainfluenzae*	*H. parainfluenzae*	1
	*H. parainfluenzae*	*H. parainfluenzae*	**No I.D.**	***H. influenzae***	-	1
*H. haemolyticus*	*H. haemolyticus* ****(both probes)	***H. influenzae***	***H. influenzae***	-	-	1
	***H. influenzae*** ** (one out of two probes)**, *H. haemolyticus* ****(both probes)	***H. influenzae***	***H. influenzae***	-	-	1
	*H. haemolyticus* ****(both probes)	***H. influenzae***	***H. influenzae***	-	***H. parainfluenzae***	1
No sequencing performed****(*H. haemolyticus* ATCC 33390, 16N, 27p25, PN24)	*H. haemolyticus* ****(both probes)	***H. influenzae***	*H. haemolyticus*	-	-	3
	*H. haemolyticus* ****(both probes)	***H. influenzae***	***H. influenzae***	-	-	1
*H. parahaemolyticus*	***H. influenzae (one out of two probes)***	*H. parahaemolyticus*	***H. influenzae***	-	-	1

Sequencing of the 16S rRNA gene of clinical *Haemophilus* spp. isolates and strains from strain collections was done if the best match for any of the diagnostic procedures performed led to misleading or contradictory results. The well-characterized *H. haemolyticus* strains that were donated by the University of Aarhus were not sequenced. (“No ID” indicates that the diagnostic procedure did not even allow for identification to genus level).

**Bold type** indicates misleading or contradictory diagnostic results. For clarity, the table presents only best matches without percentages indicating the reliability of each diagnostic result.

## Discussion

Discrimination of the closely related species *Haemophilus influenzae*, *H. parainfluenzae*, and *H. haemolyticus* is of diagnostic importance because of their striking differences in pathogenicity [2], [6]–[8], [10]. Although highly sophisticated molecular procedures for the reliable discrimination of *H. haemolyticus* und *H. influenzae* have been described [7], they are hardly suitable for use under routine diagnostic conditions. These procedures demonstrated frequent occurrence of commensal *H. haemolyticus* and misidentification as pathogenic *H. influenzae* [7], [27], also observed in our study. A reliable identification of *H. haemolyticus* and discrimination from pathogenic *H. influenzae* is important for diagnostic and epidemiological purposes.

However, our results confirm that classical biochemical identification usually fails to identify this agent to species level. The formerly proposed discrimination of *H. influenzae* from *H. haemolyticus* by virtue of prominent beta-hemolysis of the latter, preferably on horse blood agar [44]–[46], allowing discrimination by simple macroscopic inspection of the culture medium, is not reliable because non-hemolytic *H. haemolyticus* isolates occur frequently [7], [27]. Also in our study, all three clinical isolates from Rostock and Ulm were misidentified by biochemical/morphological methods. *H. haemolyticus*-induced hemolysis only works with supplements of horse, bovine, or rabbit blood but not with the more frequently used sheep blood supplement [45]. Thus, reliable routine-compatible diagnostic procedures for the discrimination of the two species are desirable.

Accordingly, the study's objective was to compare FISH and two different MALDI-TOF-MS platforms in terms of their ability to discriminate *H. influenzae* from the comparatively frequent commensal bacteria *H. parainfluenzae* and *H. haemolyticus*. Other pathogenic *Haemophilus* spp. such as *Haemophilus influenzae* biogroup *aegyptius* [47] were not included in the study because of their known rarity in Western Europe.

The study may be biased by the fact that the results of the Shimadzu MALDI-TOF-MS procedure, one of the procedures compared, were part of the inclusion criteria at least for the clinical isolates from Rostock. Clinical strains which might erroneously have not been identified as *Haemophilus* spp. were thus *a priori* excluded from further analyses. Consequently, our results might tend to overestimate the percentage of correct Shimadzu MALDI-TOF-MS results. This bias was unavoidable because Shimadzu MALDI-TOF-MS was already the method of first choice for bacterial identification in the diagnostic routine in Rostock when the study was performed. Another source of bias might be the fact that no blinding was performed. Each study participant had *ad libitum* access to the main database where all test results were documented. In particular, the subjective scoring criteria of FISH might have been affected by the bias due to lack of blinding. However, sequencing was not performed before all other diagnostic results were present, so each investigator was unaware of the definite strain identities. *H. haemolyticus*, in particular, was correctly identified by FISH in spite of contradictory MALDI-TOF-MS results. More than one-third of all tested clinical isolates had to be sequenced due to contradictory test results, suggesting that the investigators were at least not severely influenced by the lack of blinding. Nevertheless, we admit that slightly poorer results might have been observed if blinding had been implemented.

For rapid therapeutic decisions, distinction of *H. influenzae* and *H. parainfluenzae* is the most important task due to their high frequency of isolation and highly similar colony morphology on agar plates but clear-cut different clinical relevance. Identification of *H. influenzae* was achieved by the combined use of the newly developed FISH probes with 86% (43/50) reliability, for *H. parainfluenzae* with 84% (21/25) reliability, and for *H. haemolyticus* with 86% (6/7) reliability. Altogether 15.5% of failed identifications were observed for the combined use of all newly elaborated *Haemophilus* probes with *Haemophilus* spp. strains, resulting in uninterpretable results but no misidentifications. To avoid misidentifications due to missed or non-specific bindings of one single probe, identification of *H. influenzae* should only be accepted if both Hain 16S1251 and Hain 16S1253 + Hain 16S1253comp show specific binding. A reduced sensitivity, as observed for our newly designed probe/competitor probe combination Hain 16S1253 + Hain 16S1253comp, was also seen for the previously published *H. influenzae/H. haemolyticus* probe [22]: when we tested this probe with 50 *H. influenzae* strains in a pilot study (data not shown), it missed five strains (10%) as expected from a prior data analysis in GenBank (http://blast.ncbi.nlm.nih.gov), which demonstrated considerable sequence variability of various *H. influenzae* strains at the binding site of the probe (data not shown). The two *H. haemolyticus* probes of our study should be used in parallel assays as well, because at least one will specifically show no binding in case of a non-target organism, while the other will at most display unspecific borderline fluorescence intensities. In any case, all the *Haemophilus* probes described here should be used in concert, since cross-reactivity might theoretically occur in any direction and contradictory FISH reactions would lead to uninterpretable results rather than misidentifications.

FISH-based diagnosis of *Haemophilus* spp. to species level is challenging because of close sequence homology of ribosomal RNA [7], [27]. Alternative targets, including RNA of the ribosomal protein beta subunit (*rpoB*) gene, which can be used for sequence-based identification to species [42] or clonal [48] level, are less suitable for FISH. Ribosomes, i.e., ribosomal RNA, are usually more numerous within viable bacteria than RNA of other targets, making 16S or 23S ribosomal RNA particularly useful for FISH by providing optimum fluorescence intensity [36]. As expected from the *in-silico* evaluation, the observed cross-reactions affected the probes with intended specificity for *H. influenzae* and *H. haemolyticus* but not the probe for *H. parainfluenzae*. The genetic background of lacking or incorrect binding to individual strains was not assessed, because the binding sites of the respective FISH probes were beyond the amplification range of the 16S rRNA gene PCR used. In spite of the observed problems with individual isolates, the multiple-probe assay described here correctly identified 85% of the strains and led to no misidentifications in spite of a few failed identifications to species level. Uninterpretable results still have to be ruled out by additional approaches.

Among these additional approaches, Bruker MALDI-TOF-MS with labor-intensive formic-acid extraction as suggested by the manufacturer correctly identified 100% of the tested *H. influenzae* strains. However, 4 (11.8%) failed identifications and 6 (17.6%) misidentifications were observed for the 34 other *Haemophilus* spp. strains in a combined analysis with and without the extraction procedure. Although omitting the extraction did not lead to significant differences in the mean identification scores, identification to species level would have failed without it for 6 of 84 *Haemophilus* strains (7.1%). The misidentifications were exclusively associated with the lack of a *H. haemolyticus* reference spectrum in the database. With the addition of an appropriate in-house reference spectrum, the procedure constituted a reliable tool for the discrimination of *H. influenzae*, *H. parainfluenzae*, and *H. haemolyticus*. However, the fact that 12% of the analyzed *H. influenzae* strains matched our newly designed *H. haemolyticus* reference spectrum with identification scores of >2.0 stresses the great spectral similarity of *H. influenzae* and *H. haemolyticus*, making a MALDI-TOF-MS-based discrimination challenging. Nevertheless, we recommend the implementation of *H. haemolyticus* reference spectra, e.g., based on the ATCC 33390 *H. haemolyticus* reference strain, in the manufacturer's database to allow for broader evaluation in the diagnostic routine setting. In spite of the fact that 12% of the analyzed *H. influenzae* strains matched our newly designed *H. haemolyticus* reference spectrum with identification scores of >2.0, all of these *H. influenzae* strains showed better matching with *H. influenzae* spectra, leading to no misidentifications and distinguishable clustering of *H. influenzae* and *H. haemolyticus* spectra.

In a previously described study applying Bruker MALDI-TOF-MS technology to phylogenetically related bacterial species, Couturier *et al*. detected 93% correct identifications of HACEK (*H. parainfluenzae*, *Aggregatibacter actinomycetemcomitans*, *A. aphrophilus*, *Cardiobacterium hominis*, *Eikenella corrodens*, *Kingella kingae*), and *H. influenzae* clinical isolates to genus levels. However, no *H. haemolyticus* strains were included [49]. The group of van Veen *et al*. showed correct identification results of 84% of the analyzed strains among a group they called “‘miscellaneous bacteria,” including 51 HACEK organisms, and *H. influenzae*, with the Bruker system. Among the HACEK organisms, 98% were correctly identified to the species level, while one *H. parainfluenzae* isolate could only be differentiated to the genus level by MALDI-TOF-MS [50]. Our results of 88% correct identifications to species level using Bruker MALDI-TOF-MS technology fit well with the previously published data, considering that the authors of the previous works did not intentionally include *H. haemolyticus* isolates. Earlier investigations, including Haag *et al*.'s proof-of-principle analysis of the suitability of MALDI-TOF-MS for discrimination within the *Haemophilus* genus using a PerSeptive Biosystems (Framingham, MA, USA) Voyager-DE MALDI-TOF-MS mass spectrometer, are not directly comparable due to the use of different experimental settings. Haag *et al.* demonstrated general differences between MALDI-TOF-MS spectra of *H. influenzae*, *H. parainfluenzae*, *Haemophilus aphrophilus*, and *Haemophilus ducreyi*. However, they did not perform evaluations with considerable numbers of strains [26].

Shimadzu MALDI-TOF-MS analysis after straightforward use of colony material led to considerably worse results, with 22.6% failed identifications and 7.1% misidentifications. At least 70% of the *H. haemolyticus* strains would not have been misidentified as *H. influenzae* due to low identification scores. A possibility of the correct species identity was indicated for only 43% of the *H. haemolyticus* strains, confirming the close similarity of *H. influenzae* and *H. haemolyticus* spectra. Speed and ease of handling are the major advantages of MALDI-TOF-MS analysis without prior extraction, so it could be useful for a rapid diagnostic approach if low identification scores are regularly resolved by alternative methods.

In contrast to previous studies, in which biochemical methods, i.e., API NH and VITEK 2 NH, led to discrepancy rates as low as 1% to 10% [15], [17] among *Haemophilus* spp., the results of biochemical identification, particularly with the API NH system, were surprisingly poor with nearly 20% failed or incorrect identifications in spite of its being performed by experienced technicians of an accredited laboratory. A simultaneous dense culture of mixed *Haemophilus* spp. could be one explanation, since co-colonization of *H. influenzae* and *H. parainfluenzae* on nasal mucosa has been shown to occur *in situ* by a multiplex PCR approach [51]. In this study, the PCR-based detection rate was 44.14% (294/666) for *H. influenzae* and 61.26% (408/666) for *H. parainfluenzae* in nasopharyngeal swab specimens, suggesting co-colonizations for at least 5.41% (36/666) of the samples [51].

None of the diagnostic procedures described here correctly identified all *Haemophilus* strains to species level without any failed identifications or misidentifications. However, bearing their individual weaknesses in mind, each has its clear value in diagnostic algorithms for *Haemophilus* spp. identification.

Due to the simple execution, the short performance time of only 1.5 hours, and the low risk of misidentifications, FISH with the probes described here may be used for the discrimination of *H. influenzae*, *H. parainfluenzae*, and *H. haemolyticus* in routine laboratories if rapid results are desired, resources are scarce, and sophisticated molecular methods are not available. Costing less than one euro per analysis including all required probes, FISH is considerably less expensive than commercial biochemical tests and most other molecular methods. The technique requires no more than standard equipment of a good microbiological diagnostic laboratory, including an incubator and a fluorescence microscope. Nevertheless, in case of uninterpretable results, alternative diagnostic approaches have to be considered. Sequencing will usually be reserved for very rare life-threatening situations or forensic needs if severe consequences of misidentifications are to be anticipated. As sequencing is still too expensive and time-consuming for the diagnostic routine, biochemical identification and MALDI-TOF-MS technology will be the most commonly chosen alternatives to FISH, although they may lead to misidentifications as well in 10–20% of the cases as shown in this study. Among the 13 *Haemophilus* spp. strains with inconclusive FISH results, there was one misidentification by Bruker MALDI-TOF-MS, and there were 4 failed identifications or incorrect best matches by Shimadzu MALDI-TOF-MS.

After addition of an appropriate *H. haemolyticus* reference spectrum, Bruker MALDI-TOF-MS with prior formic acid extraction provided the best diagnostic results. However, the formic acid extraction is quite laborious, limiting its use for the routine laboratory. The superiority of the discriminatory performance of MALDI-TOF-MS to that of FISH deteriorates when colony material is used directly from the culture medium, as was confirmed for the rapid Shimadzu MALDI-TOF-MS procedure. Yet there could be a net advantage of this procedure due its very low hands-on time in conjunction with the few occasions when subsequent alternative tests are used to resolve uninterpretable identification scores. Although the costs-per-analysis for MALDI-TOF-MS are also low, the considerable costs of system acquisition might limit the technique's implementation in a resource-limited setting.

Given the diagnostic weaknesses shown for all the available procedures, the correct discrimination of closely related *Haemophilus* spp. with rapid routine methods remains challenging. Alternative diagnostic methods should be available in an algorithm for routine diagnostic laboratories if results are uninterpretable and clear results should still be carefully interpreted in conjunction with the clinical status of the patient.

## Supporting Information

Table S1 **Partial 16S rRNA gene sequences.** Sequences of 16S rRNA gene fragments as obtained from 30 *Haemophilus* spp. strains, for which FISH, MALDI-TOF-MS, or biochemical identification led to misleading or inconclusive results. In a few instances, short readable sequences had to be accepted if the die-off of the respective isolates after freezing did not allow for new cultural growth with consecutive repeated PCR and sequencing.
(DOC)
