# Supplementary material for: Rapid Discrimination of Haemophilus influenzae, H. parainfluenzae, and H. haemolyticus by Fluorescence In Situ Hybridization (FISH) and Two Matrix-Assisted Laser-Desorption-Ionization Time-of-Flight Mass Spectrometry (MALDI-TOF-MS) Platforms
Source: PLoS One. 2013 Apr 30;8(4):e63222. doi: 10.1371/journal.pone.0063222 (PMC3639997; doi:10.1371/journal.pone.0063222)
Supplement: Table S1 — Partial 16S rRNA gene sequences. Sequences of 16S rRNA gene fragments as obtained from 30 Haemophilus spp. strains, for which FISH, MALDI-TOF-MS, or biochemical identification led to misleading or inconclusive results. In a few instances, short readable sequences had to be accepted if the die-off of the respective isolates after freezing did not allow for new cultural growth with consecutive repeated PCR and sequencing. (DOC) [file pone.0063222.s001.doc]

**Table S1. Partial 16S rRNA gene sequences.** Sequences of 16S rRNA gene fragments as obtained from 30 *Haemophilus* spp. strains, for which FISH, MALDI-TOF-MS, or biochemical identification led to misleading or inconclusive results. In a few instances, short readable sequences had to be accepted if the die-off of the respective isolates after freezing did not allow for new cultural growth with consecutive repeated PCR and sequencing.

| **16S rRNA gene fragments of clinical *H. influenzae* strains (n = 14)** |
| --- |
| >110353  TCCGTTAGCTACGGGCGCCAGAGTTAAACCCCAACCCCCAAATCGACAGCGTTTACAGCGTGGACTACCAGGGTATCTAATCCTGTTTGCTCCCCACGCTTTCGCACATGAGCGTCAGTACATTCCCAAGGGGCTGCCTTCGCCTTCGGTATTCCTCCACATCTCTACGCATTTCACCGCTACACGTGGAATTCTACCCCTCCCTAAAGTACTCTAGTTACCCAGTCTGAAATGCAATTCCCAGGTTAAGCCCGGGGCTTTCACACCTCACTTAAATAACCGCCTGCGTGCCCTTTACGCCCAGTTATTCCGATTAACGCTCGCACCCTCCGTATTACCGCGGCTGCTGGCACGGAGTTAGCCGGTGCTTCTTCTGTATTTAACGTCAATTTGATGTGCTATTAACACATCAACCTTCCTCAATACCGAAAGAACTTTACAACCCGAAGGCCTTCTTCATTCACGCGGCATGGCTGCGTCAGGGTTCCCCCCATTGCGCAATATNCCCCACTGCTGCCTCCCGTAGGAGTCTGGACCGNGTCTCAGTTCCAGTGTGSCTGGNCATCCTCTCAGACCAGCTAGAGATCGCAGGCTTGGTAGGCATTTACCCCACCAACTACCTAATCCCACTTGGGCTCATCCTATGGCATGCGGCCTCTCAGTCCCGCACTTTCATCTTCCGATAATACGCGGTATTAGCGACAGTTTCCCGTCGTTATCCCCCTCCATAAGCCAGATTCCCAAGCATTACTCACCCGTCCGCCACTCGTCAGCAAGAAAGCAAGCTTTCTCCTGCTACCGTTCGACTGGC |
| >189053  GATGAGCCCAAGTGGGATTAGGTAGTTGGTGGGGTAAATGCCTACCAAGCCTGCGATCTCTAGCTGGTCTGAGAGGATGACCAGCCACACTGGAACTGAGACACGGTCCAGACTCCTACGGGAGGCAGCAGTGGGGAATATTGCGCAATGGGGGGAACCCTGACGCAGCCATGCCGCGTGAATGAAGAAGGCCTTCGGGTTGTAAAGTTCTTTNGGTATTGAGGAAGGTTGATGTGTTAATAGCACATCAAATTGACGTTAAATACAGAAGAAGCACCGGCTAACTCCGTGCCAGCAGCCGCGGTAATACGGAGGGTGCGAGCGTTAATCGGAATAACTGGGCGTAAAGGGCACGCAGGCGGTTATTTAAGTGAGGTGTGAAAGCCCCGGGCTTAACCTGGGAATTGCATTTCAGACTGGGTAACTAGAGTACTTTAGGGAGGGGTAGAATTCCACGTGTAGCGGTGAAATGCGTAGAGATGTGGAGGAATACCGAAGGCGAAGGCAGCCCCTTGGGAATGTACTGACGCTCATGTGCGAAAGCGTGGGGAGCAAACAGGATTAGATACCCTGGTAGTCCACGCTGTAAACGCTGTCGATTTGGGGGTTGGGGTTTAACTCTGGCGCCCGTAGCTAACGTGATAAATCGA |
| >v00271  ATCCGTTAGCTACGGGCGCCAGAGTTAAACCCCAACCCCCAAATCGACAGCGTTTACAGCGTGGACTACCAGGGTATCTAATCCTGTTTGCTCCCCACGCTTTCGCACATGAGCGTCAGTACATTCCCAAGGGGCTGCCTTCGCCTTCGGTATTCCTCCACATCTCTACGCATTTCACCGCTACACGTGGAATTCTACCCCTCCCTAAAGTACTCTAGTTACCCAGTCTGAAATGCAATTCCCAGGTTAAGCCCGGGGCTTTCACACCTCACTTAAATAACCGCCTGCGTGCCCTTTACGCCCAGTTATTCCGATTAACGCTCGCACCCTCCGTATTACCGCGGCTGCTGGCACGGAGTTAGCCGGTGCTTCTTCTGTATTTAACGTCAATTTGATGTGCTATTAACACATCAACCTTCCTCAATACCGAAAGAACTTTACAACCCTAAGGCCTTCTTCATTCACGCGGCATGGCTGCGTCAGGGTTCCCCCCATTGCGCAATATTCCCCACTGCTGCCTCCCGTAGGAGTCTGGACCGTGTCTCAGTTCCAGTGTGGCTGGTCATCCTCTCAGACCAGCTAGAGATCGCAGGCTTGGTAGGCYTTTACCCCACCAACTACCTAATCCCACTTGGGCTCATCCTATGGCATGTGGCCTGNAGGTCCCGCACTTTAATCTTTCGATANTACGCGGTATTAGCGACAGTTTCCCGTCGTTATCCCCCTCCATAAGCCAGATTCCCAAGCATTACTCACCCGTCCGCCACTCGTCAGCAAGAAAGCAAGCTTTCTCCTGCTACCGTTCGACTTG |
| >v00367  TCCGTTAGCTACGGGCGCCAGAGTTAAACCCCAACCCCCAAATCGACAGCGTTTACAGCGTGGACTACCAGGGTATCTAATCCTGTTTGCTCCCCACGCTTTCGCACATGAGCGTCAGTACATTCCCAAGGGGCTGCCTTCGCCTTCGGTATTCCTCCACATCTCTACGCATTTCACCGCTACACGTGGAATTCTACCCCTCCCTAAAGTACTCTAGTTACCCAGTCTGAAATGCAATTCCTAGGTTAAGCCCAGGGCTTTCACACCTCACTTAAATAACCGCCTGCGTGCCCTTTACGCCCAGTTATTCCGATTAACGCTCGCACCCTCCGTATTACCGCGGCTGCTGGCACGGAGTTAGCCGGTGCTTCTTCTGTATTTAACGTCAATTTGATGTGCTATTAACACATCAACCTTCCTCAATACCGAAAGAACTTTACAACCCTAAGGCCTTCTTCATTCACGCGGCATGGCTGCGTCAGGGTTCCCCCCATTGCGCAATATTCCCCACTGCTGCCTCCCGTAGGAGTCTGGACCGTGTCTCAGTTCCAGTGTGGCTGGTCATCCTCTCAGACCAGCTAGAGATCGCAGGCTTGGTAGGCCTTTACCCCACCAACTACCTAATCCCACTTGGGCTCATCCTATGGCATGCGGCCTCTCAGTCCCGCACTTTCATCTTCCGATAATACGCGGTATTAGCGACAGTTTCCCGTCGTTATCCCCCTCCATAAGCCAGATTCCCAAGCATTACTCACCCGTCCGCCACTCGTCAGCAAGAAAGCAAGC |
| >v00370  TCGATTTCCGTTAGCTACGGGCGCCAGAGTTAAACCCCAACCCCCAAATCGACAGCGTTTACAGCGTGGACTACCAGGGTATCTAATCCTGTTTGCTCCCCACGCTTTCGCACATGAGCGTCAGTACATTCCCAAGGGGCTGCCTTCGCCTTCGGTATTCCTCCACATCTCTACGCATTTCACCGCTACACGTGGAATTCTACCCCTCCCTAAAGTACTCTAGTTACCCAGTCTGAAATGCAATTCCTAGGTTAAGCCCAGGGCTTTCACACCTCACTTAAATAACCGCCTGCGTGCCCTTTACGCCCAGTTATTCCGATTAACGCTCGCACCCTCCGTATTACCGCGGCTGCTGGCACGGAGTTAGCCGGTGCTTCTTCTGTATTTAACGTCAATTTGATGTGCTATTAACACATCAACCTTCCTCAATACCGAAAGAACTTTACAACCCTAAGGCCTTCTTCATTCACGCGGCATGGCTGCGTCAGGGTTCCCCCCATTGCGCAATATTCCCCACTGCTGCCTCCCGTAGGAGTCTGGACCGTGTCTCAGTTCCAGTGTGGCTGGTCATCCTCTCAGACCAGCTAGAGATCGCAGGCTTGGTAGGCCTTTACCCCACCAACTACCTAATCCCACTTGGGCTCATCCTATGGCATGCGGCCTCTCAGTCCCGCACTTTCATCTTCCGATAATACGCGGTATTAGCGACAGTTTCCCGTCGTTATCCCCCTCCATAAGCCAGATTCCCAAGCATTACTCACCCGTCCGCCACTCGTCAGCAAGAAAGCAAGCTTTCTCCTGCTACCGTTCGACTTGCA |
| >v00388  TCGATTTTCCGTTAGCTACGGGCGCCAGAGTTAAACCCCAACCCCCAAATCGACAGCGTTTACAGCGTGGACTACCAGGGTATCTAATCCTGTTTGCTCCCCACGCTTTCGCACATGAGCGTCAGTACATTCCCAAGGGGCTGCCTTCGCCTTCGGTATTCCTCCACATCTCTACGCATTTCACCGCTACACGTGGAATTCTACCCCTCCCTAAAGTACTCTAGTTACCCAGTCTGAAATGCAATTCCNAGGTTAAGCCCRGGGCTTTCACACCTCACTTAAATAACCGCCTGCGTGCCCTTTACGCCCAGTTATTCCGATTAACGCTCGCACCCTCCGTATTACCGCGGCTGCTGGCACGGAGTTAGCCGGTGCTTCTTCTGTATTTAACGTCAATTTGATGTGCTATTAACACATCAACCTTCCTCAATACCGAAAGAACTTTACAACCCTAAGGCCTTCTTCATTCACGCGGCATGGCTGCGTCAGGGTTCCCCCCATTGCGCAATATTCCCCACTGCTGCCTCCCGTAGGAGTCTGGACCGTGTCTCAGTTCCAGTGTGGCTGGTCATCCTCTCAGACCAGCTAGAGATCGCAGGCTTGGTAGGCCTTTACCCCACCAACTACCTAATCCCACTTGGGCTCATCCTATGGCATGCGGCCTCTCAGTCCCGCACTTTCATCTTCCGATAATACGCGGTATTAGCGACAGTTTCCCGTCGTTATCCCCCTCCATAAGCCAGATTCCCAAGCATTACTCACCCGTCCGCCACTCGTCAGCAAGAAAGCAAGCTTTCTCCTGCTACCGTTCGACTTGC |
| >v00549  TCGATTTATCACGTTAGCTACGGGCGCCAGAGTTAAACCCCAACCCCCAAATCRACAGCGTTTACAGCGTGGACTACCAGGGTATCTAATCCTGTTTGCTCCCCACGCTTTCGCACATGAGCGTCAGTACATTCCCAAGGGGCTGCCTTCGCCTTCGGTATTCCTCCACATCTCTACGCATTTCACCGCTACACGTGGAATTCTRCCCCTCCCTAAAGTACTCTAGTTACCCAGTCTGAAATGCWATTCCCAGGTTAAGCCCGGGGCTTTCACACCTCACTTAAATAACCGCCTGCGTGCCCTTTACGCCCAGTTATTCCGATTAACGCTCGCACCCTCCGTATTACCGCGGCTGCTGGCACGGAGTTAGCCGGTGCTTCTTCTGTATTTAACGTCAATTTGATGTACTATTAACACATCAACCTTCCTCAATACCGAAAGAACTTTACAACCCGAAGGCCTTCTTCATTCACGCGGCATGGCTGCGTCAGGGTTCCCCCCATTGCGCAATATTCCCCACTGCTGCCTCCCGTAGGAGTCTGGACCGTGTCTCAGTTCCAGTGTGGCTGGTCATCCTCTCAGACCAGCTAGAGATCGCAGGCTTGGTGAGCCATTACCCCACCAACTACCTAATCCCACTTGGGCTCATCTTATGGCATGCGGCTTTACAGTCCCGCACTTTCGTCTCTNGACACTACGNGGTATTAGCGACAGTTTCCCGTCGTTATCCCCCTCCATAAGCCAGATTCCCAAGCATTACTCACCCGTCCGCCACTCGTCAGCAAGAAAGCAAGCTTCCTCCTGCTACCGTTCGACTGCA |
| >v02130  TGCAGTCGACGGTAGCAGGAGAAAGCTTGCTTTCTTGCTGACGAGTGGCGGACGGGTGAGTAATGCTTGGGAATCTGGCTTATGGAGGGGGATAACGACGGGAAACTGTCGCTAATACCGCGTATTATCGGAAGATGAAAGTGCGGGACTGAGAGGCCGCATGCCATAGGATGAGCCCAAGTGGGATTAGGTAGTTGGTGGGGTAAATGCCTACCAAGCCTGCGATCTCTAGCTGGTCTGAGAGGATGACCAGCCACACTGGAACTGAGACACGGTCCAGACTCCTACGGGAGGCAGCAGTGGGGAATATTGCGCAATGGGGGGAACCCTGACGCAGCCATGCCGCGTGAATGAAGAAGGCCTTCGGGTTGTAAAGTTCTTTNGGTATTGAGGAAGGTTGATGTGTTAATAGCACATCAAATTGACGTTAAATACAGAAGAAGCACCGGCTAACTCCGTGCCAGCAGCCGCGGTAATACGGAGGGTGCGAGCGTTAATCGGAATAACTGGGCGTAAAGGGCACGCAGGCGGTTATTTAAGTGAGGTGTGAAAGCCCTGGGCTTAACCTAGGAATTGCATTTCAGACTGGGTAACTAGAGTACTTTAGGGAGGGGTAGAATTCCACGTGTAGCGGTGAAATGCGTAGAGATGTGGAGGAATACCGAAGGCGAAGGCAGCCCCTTGGGAATGTACTGACGCTCATGTGCGAAAGCGTGGGGAGCAAACAGGATTAGATACCCTGGTAGTCCACGCTGTAAACGCTGTCGATTTGGGGGTTGGGGTTTAACTCTGGCGCCCGTAGCTAACGTGATA |
| >v09757  TCGATTTATCACGTTAGCTACGGGCGCCAGAGTTAAACCCCAACCCCCAAATCGACAGCGTTTACAGCGTGGACTACCAGGGTATCTAATCCTGTTTGCTCCCCACGCTTTCGCACATGAGCGTCAGTACATTCCCAAGGGGCTGCCTTCGCCTTCGGTATTCCTCCACATCTCTACGCATTTCACCGCTACACGTGGAATTCTACCCCTCCCTAAAGTACTCTAGTTACCCAGTCTGAAATGCAATTCCCAGGTTAAGCCCGGGGCTTTCACACCTCACTTAAATAACCGCCTGCGTGCCCTTTACGCCCAGTTATTCCGATTAACGCTCGCACCCTCCGTATTACCGCGGCTGCTGGCACGGAGTTAGCCGGTGCTTCTTCTGTATTTAACGTCAATTTGATGTGCTATTAACACATCAACCTTCCTCAATACCGAAAGAACTTTACAACCCTAAGGCCTTCTTCATNCNCGNGGCATGGNNGNGTCAGGGTTCCCCCCATTSCNCAATATTCCCCACYGCTSCCTCCCGTANGAGTCTGGACCGTGTCTCAGNTCCAGTGNGGCTGGNCATCCTCTCAGACCASCTAGAGATCGCAGGCTTGGTAGGCYTTTACCCCACCAACTACCTAATCCCACTTGGGCTCATCCTATGGCATGYGGCCYSWNNGTCCCRCACTTTNATCTTNCGATANTACGCGGTATTAGCGACAGTTTCCCGTCGTTATCCCCCTCCATAAGCCAGATTCCCAAGCATTACTCACCCGTCCGCCACTCGTCAGCAAGAAAGCAAGCTTTCTCCTGCTACCGTCGACTGCA |
| >v09961  AGGAAGGTGGATGTTTTAATAACACATCAAATTGACGTTAAATACAGAAGAAGCACCGGCTAACTCCGTGCCAGCAGCCGCGGTAATAAGGAGGGTGCGAGCGTTAATCGGAATAACTGGGCGTAAAGGGCACGCAGGCGGTTATTTAAGTGAGGTGTGAAAGCCCTGGGCTTAACCTAGGAATTGCATTTCAGACTGGGTAACTAGAGTACTTTAGGGAGGGGTAGAATTCCACGTGTAGCGGTGAAATGCGTAGAGATGTGGAGGAATACCGAAGGCGAAGGCAGCCCCTTGGGAATGTACTGACGCTCATGTGCGAAAAGCGTGGGGAGCAAACACGGATTATGATACCCTGGTAGTGCCACCGCTGTAAACGACTGTGCGACTTTGGGGGTTGGGGTTTAAGCTCCTGGCGCCCGTAGGCCTAAACGTGATA |
| >v12394  TCGATTTTCCGTTAGCTACGGGCGCCAGAGTTAAACCCCAACCCCCAAATCGACAGCGTTTACAGCGTGGACTACCAGGGTATCTAATCCTGTTTGCTCCCCACGCTTTCGCACATGAGCGTCAGTACATTCCCAAGGGGCTGCCTTCGCCTTCGGTATTCCTCCACATCTCTACGCATTTCACCGCTACACGTGGAATTCTACCCCTCCCTAAAGTACTCTAGTTACCCAGTCTGAAATGCAATTCCNAGGTTAAGCCCRGGGCTTTCACACCTCACTTAAATAACCGCCTGCGTGCCCTTTACGCCCAGTTATTCCGATTAACGCTCGCACCCTCCGTATTACCGCGGCTGCTGGCACGGAGTTAGCCGGTGCTTCTTCTGTATTTAACGTCAATTTGATGTGCTATTAACACATCAACCTTCCTCAATACCGAAAGAACTTTACAACCCTAAGGCCTTCTTCATTCACGCGGCATGGCTGCGTCAGGGTTCCCCCCATTGCGCAATATTCCCCACTGCTGCCTCCCGTAGGAGTCTGGACCGTGTCTCAGTTCCAGTGTGGCTGGTCATCCTCTCAGACCAGCTAGAGATCGCAGGCTTGGTAGGCCTTTACCCCACCAACTACCTAATCCCACTTGGNCTCATCCTATGGCATGCGGCCTCTCAGTCCCGCACTTTCATCTTCCGATAATACGCGGTATTAGCGACAG |
| >v12400  TCGATTTATCACGTTAGCTACGGGCGCCAGAGTTAAACCCCAACCCCCAAATCGACAGCGTTTACAGCGTGGACTACCAGGGTATCTAATCCTGTTTGCTCCCCACGCTTTCGCACATGAGCGTCAGTACATTCCCAAGGGGCTGCCTTCGCCTTCGGTATTCCTCCACATCTCTACGCATTTCACCGCTACACGTGGAATTCTACCCCTCCCTAAAGTACTCTAGTTACCCAGTCTGAAATGCAATTCCCAGGTTAAGCCCGGGGCTTTCACACCTCACTTAAATAACCGCCTGCGTGCCCTTTACGCCCAGTTATTCCGATTAACGCTCGCACCCTCCGTATTACCGCGGCTGCTGGCACGGAGTTAGCCGGTGCTTCTTCTGTATTTAACGTCAATTTGATGTACTATTAACACATCAACCTTCCTCAATACCGAAAGAACTTTACAACCCGAAGGCCTTCTTCATTCACGCGGCATGGCTGCGTCAGGGTTCCCCCCATTGCGCAATATTCCCCACTGCTGCCTCCCGTAGGAGTCTGGACCGTGTCTCAGTTCCAGTGTGGCTGGTCATCCTCTCAGACCAGCTAGAGATCGCAGGCTTGGTGAGCCTTTACCCCACCAACTACCTAATCCCACTTGGGCTCATCCTATGGCAGGTGGCTAAAAGTCCCACCCTTTCGTCTCTCGACACTACGCGGTATTAGCGACAGTTTCCCGTCGTTATCCCCCTCCATAAGCCAGATTCCCAAGCATTACTCACCCGTCCGCCACTCGTCAGCAAGAAAGCAAGCTTCCTCCTGCTACCGTCGACTGCA |
| >v12424  TCGATTTTCCGTTAGCTACGGGCGCCAGAGTTAAACCCCAACCCCCAAATCGACAGCGTTTACAGCGTGGACTACCAGGGTATCTAATCCTGTTTGCTCCCCACGCTTTCGCACATGAGCGTCAGTACATTCCCAAGGGGCTGCCTTCGCCTTCGGTATTCCTCCACATCTCTACGCATTTCACCGCTACACGTGGAATTCTACCCCTCCCTAAAGTACTCTAGTTACCCAGTCTGAAATGCAATTCCCAGGTTAAGCCCGGGGCTTTCACACCTCACTTAAATAACCGCCTGCGTGCCCTTTACGCCCAGTTATTCCGATTAACGCTCGCACCCTCCGTATTACCGCGGCTGCTGGCACGGAGTTAGCCGGTGCTTCTTCTGTATTTAACGTCAATTTGATGTGCTATTAACACATCAACCTTCCTCAATACCGAAAGAACTTTACAACCCTAAGGCCTTCTTCATTCACGCGGCATGGCTGCGTCAGGGTTCCCCCCATTGCGCAATATTCCCCACTGCTGCCTCCCGTAGGAGTCTGGACCGTGTCTCAGTTCCAGTGTGGCTGGTCATCCTCTCAGACCAGCTAGAGATCGCAGGCTTGGTAGGCYTTTACCCCACCAACTACCTAATCCCACTTGGGCTCATCCTATGGCATGNGGCCNNNNNGTCCCNCACTTTNATCTTYNGATANTACGCGGTATTAGCGACAGTTTCCSGTCGTTATCCCCCTCCATAAGCCAGATTCCCAAGCATTACTCACCCGTCCGCCACTCGTCAGCAAGAAAGCAAGCTTTCTCCTGCTACCGTTCGACTGCA |
| >v12489  TCGATTTATCACGTTAGCTACGGGCGCCAGAGTTAAACCCCAACCCCCAAATCGACAGCGTTTACAGCGTGGACTACCAGGGTATCTAATCCTGTTTGCTCCCCACGCTTTCGCACATGAGCGTCAGTACATTCCCAAGGGGCTGCCTTCGCCTTCGGTATTCCTCCACATCTCTACGCATTTCACCGCTACACGTGGAATTCTACCCCTCCCTAAAGTACTCTAGTTACCCAGTCTGAAATGCAATTCCCAGGTTAAGCCCGGGGCTTTCACACCTCACTTAAATAACCGCCTGCGTGCCCTTTACGCCCAGTTATTCCGATTAACGCTCGCACCCTCCGTATTACCGCGGCTGCTGGCACGGAGTTAGCCGGTGCTTCTTCTGTATTTAACGTCAATTTGATGTGCTATTAACACATCAACCTTCCTCAATACCGAAAGAACTTTACAACCCGAAGGCCTTCTTCATTCACGCGGCATGGCTGCGTCAGGGTTCCCCCCATTGCGCAATATTCCCCACTGCTGCCTCCCGTAGGAGTCTGGACCGTGTCTCAGTTCCAGTGTGGCTGGTCATCCTCTCAGACCAGCTAGAGATCGCAGGCTTGGTAGGCATTTACCCCACCAACTACCTAATCCCACTTGGGCTCATCCTATGGCATGCGGCCTCNCAGTCCCGCACTTTCATCTTCCGATAATACGCGGTATTAGCGACAGTTTCCNGTNGTTATCCCCCTCCATAAGCCAGATTCCCAAGCATTACTCACCCGTCCGCCACTCGTCAGCAAGAAAGCAAGCTTTCTCCTGCTACCGTCGACTGCA |
| **16S rRNA gene fragments of clincial *H. parainfluenzae* strains (n = 12)** |
| >191311  AAGAGTTTGATCCTGGCTCAGATTGAACGCTGGCGGCAGGCTTAACACATGCAAGTCGAACGGTAACATAAAGAAGCTTGCTTCTTTGATGACGAGTGGCGGACGGGTGAGTAATGCTTGGGAATCTAGCTTATGGAGGGGGATAACTACGGGAAACTGTAGCTAATACCGCGTAGTATCGNAAGATGAAAGTGTGGGACCTTCGGGCCACATGCCATAGGATGAGCCCAAGTGGGATTAGGTAGTTGGTGAGGTAAAGGCTCACCAAGCCGACGATCTCTAGCCTGGTCTGAGAGG |
| >v00369  ATCACGTTAGCTACGGGCGCCAARCTTAAAGTTCAACCCCCAAATCGACATCGTTTANAGCGTGGACTACCAGGGTATCTAATCCTGTTTGCTCCCCACGCTTTCGCACATGAGCGTCAGTACATTCCCAAGGGGCTGCCTTCGCCTTCGGTATTCCTCCACATCTCTACGCATTTCACCGCTACACGTGGAATTCTACCCCTCCCTAAAGTACTCTAGCGACCCAGTATGAAATGCAATTCCCAGGTTAAGCCCGGGGCTTTCACACCTCACTTAAGTCACCGCCTGCGTGCCCTTTACGCCCAGTTATTCCGATTAACGCTCGCACCCTCCGTATTACCGCGGCTGCTGGCACGGAGTTAGCCGGTGCTTCTTCTGTAGTTAACGTCAATCACCTAGTCTATTAAACTAAATGCCTTCCTCGCTACCGAAAGAACTTTACAACCCGAAGGCCTTCTTCATTCACGCGGCATGGCTGCGTCAGGGTTGCCCCCATTGCGCAATATTCCCCACTGCTGCCTCCCGTAGGAGTCTGGGCCGTGTCTCAGTCCCAGTGTGGCTGGTCATCCTCTCAGACCAGCTAGAGATCGTCGGCTTGGTGAGCCTTTACCTCACCAACTACCTAATCCCACTTGGGCTCATCCTATGGCATGTGGCCCGAAGGTCCCACACTTTCATCTCCNGATTCTACGCGGTATTAGCTACAGTTTCCCGTAGTTATCCCCCTCCATAAGCCAGATTCCCAAGCATTACTCACCCGTCCGCCACTCGTCATCAAAGAAGCAAGC |
| >v00650  ATCaCGTTAGCTACGGGCGCCAAGCTCAAAGCTCAACCCCCAAATCGACATCGTTTACAGCGTGGACTACCAGGGTATCTAATCCTGTTTGCTCCCCACGCTTTCGCACtTGAGCGTCAGTACATTCCCAAGGGGCTGCCTTCGCCTTCGGTATTCCTCCACATCTCTACGCATTTCACCGCTACACGTGGAATTCTACCCCTCCCTAAAGTACTCTAGCGACCCAGTATGAAATGCAATTCCCAGGTTAAGCCCGGGGCTTTCACACCTCACTTAAGTCACCGCCTGCGTGCCCTTTACGCCCAGTTATTCCGATTAACGCTCGCACCCTCCGTATTACCGCGGCTGCTGGCACGGAGTTAGCCGGTGCTTCTTCTGTAGTTAACGTCAATCAYYTAGTYTATTAAACTAAATGCCTTCCTCGCTACCGAAAGAACTTTACAACCCGAAGGCCTTCTTCATTCACGCGGCATGGCTGCGTCAGGGTTGCCCCCATTGCGCAATATTCCCCACTGCTGCCTCCCGTAGGAGTCTGGGCCGTGTCTCAGTCCCAGTGTGGCTGGTCATCCTCTCAGACCAGCTAGAGATCGTCGGCTTGGTGAGCCTTTACCTCACCAACTACCTAATCCCACTTGgGCTCATCcTATGGCATGtG |
| >v00660  GCCGCGTGAATGAAGAAGGCCTTCGGGTTGTAAAGTTCTTTCGGTAGCGAGGAAGGCATTTAGTTTAATAGACTAGGTGATTGACGTTAACTACAGAAGAAGCACCGGCTAACTCCGTGCCNGCAGCCGCGGTAATACGGAGGGTGCGAGCGTTAATNGGAATAACTGGGCGTAAAGGGCACGCAGGCGGTGACTTAAGTGAGGTGTGAAAGCCCCGGGCTTAACCTGGGAATTGCATTTCATACTGGGTCGCTAGAGTACTTTAGGGAGGGGTAGAATTCCACGTGTAGCGGTGAAATGCGTAGAGATGTGGAGGAATACCGAAGGCGAAGGCAGCCCCTTGGGAATGTACTGACGCTCATGTGCGAAAGCGTGGGGAGCAAACAGGATTAGATACCCTGGTAGTCCACGCTGTAAANGATGTCGATTTGGGGGTTGAGCTTTAAGCTTGGCGCCCGTAGCTAACGGAA |
| >v00910  GAAGCTTGCTTCTTTGATGCTGAGTGGCGGACGGGTGAGTAATGCTTGGGAATCTAGCTTATGGAGGGGGATAACTACGGGAAACTGTAGCTAATACCGCGTAATATCGAAAGATTAAAGTGTGGGACCTTCGGGCCACATGCCATAGGATGAGCCCAAGTGGGATTAGGTAGTTGGTGAGGTAAAGGCTCACCAAGCCGACGATCTCTAGCTGGTCTGAGAGGATGACCAGCCACACTGGGACTGAGACACGGCCCAGACTCCTACGGGAGGCAGCAGTGGGGAATATTGCGCAATGGGGGCAACCCTGACGCAGCCATGCCGCGTGAATGAAGAAGGCCTTCGGGTTGTAAAGTTCTTTCGGTAGCGAGGAAGGCATTTAGTTTAATAGACTAGGTGATTGACGTTAACTACAGAAGAAGCACCGGCTAACTCCGTGCCAGCAGCCGCGGTAATACGGAGGGTGCGAGCGTTAATCGGAATAACTGGGCGTAAAGGGCACGCAGGCGGTGACTTAAGTGAGGTGTGAAAGCCCCGGGCTTAACCTGGGAATTGCATTTCATACTGGGTCGCTAGAGTACTTTAGGGAGGGGTAGAATTCCACGTGTAGCGGTGAAATGCGTAGAGATGTGGAGGAATACCGAAGGCGAAGGCAGCCCCTTGGGAATGTACTGACGCTCATGTGCGAAAGCGTGGGGAGCAAACAGGATTAGATACCCTGGTAGTCCACGCTGTAAACGATGTCGATTTGGGGGTTGAACTTTGAGTTTGGCGCCCGTAGCTAACGTATA |
| >v02137  ATCACGTTTAGCTACGGGCGCCAAACCTAAAGTTCAACCCCCAAATCGACATCGTTTACAGCGTGGACTACCAGGGTATCTAATCCTGTTTGCTCCCCACGCTTTCGCACATGAGCGTCAGTACATTCCCAAGGGGCTGCCTTCGCCTTCGGTATTCCTCCACATCTCTACGCATTTCACCGCTACACGTGGAATTCTACCCCTCCCTAAAGTACTCTAGCGACCCAGTATGAAATGCAATTCCCAGGTTAAGCCCGGGGCTTTCACACCTCACTTAAGTCACCGCCTGCGTGCCCTTTACGCCCAGTTATTCCGATTAACGCTCGCACCCTCCGTATTACCGCGGCTGCTGGCACGGAGTTAGCCGGTGCTTCTTCTGTAGTTAACGTCAATCANCTAGTnTATTAAACTAAATGCCTTCCTCGCTACCGAAAGAACTTTACAACCCGAAGGCCTTCTTCATTCACGCGGCATGGCTGCGTCAGGGTTGCCCCCATTGCGCAATATTCCCCACTGCTGCCTCCCGTAGGAGTCTGGGCCGTGTCTCAGTCCCAGTGTGGCTGGTCATCCTCTCAGACCAGCTAGAGATCGTCGGCTTGGTGAGCCTTTACCTCACCAACTACCTAATCCCA |
| >v02218  AGATTGAACGCTGGCGGCAGGCTTAACACATGCAAGTCGAACGGTAACATAAAGAAGCTTGCTTCTTTGATGACGAGTGGCGGACGGGTGAGTAATGCTTGGGAATCTAGCTTATGGAGGGGGATAACTACGGGAAACTGTAGCTAATACCGCGTAATATCGAAAGATTAAAGTGTGGGACCTTCGGGCCACATGCCATAGGATGAGCCCAAGTGGGATTAGGTAGTTGGTGAGGTAAAGGCTCACCAAGCCGACGATCTCTAGCTGGTCTGAGAGGATGACCAGCCACACTGGGACTGAGACACGGCCCAGACTCCTACGGGAGGCAGCAGTGGGGAATATTGCGCAATGGGGGCAACCCTGACGCAGCCATGCCGCGTGAATGAAGAAGGCCTTCGGGTTGTAAAGTTCTTTCGGTAGCGAGGAAGGCATTTAGTTTAATAGACTAGGTGATTGACGTTAACTACAGAAGAAGCACCGGCTAACTCCGTGCCAGCAGCCGCGGTAATACGGAGGGTGCGAGCGTTAATCGGAATAACTGGGCGTAAAGGGCACGCAGGCGGTGACTTAAGTGAGGTGTGAAAGCCCCGGGCTTAACCTGGGAATTGCATTTCATACTGGGTCGCTAGAGTACTTTAGGGAGGGGTAGAATTCCACGTGTAGCGGTGAAATGCGTAGAGATGTGGAGGAATACCGAAGGCGAAGGCAGCCCCTTGGGAATGTACTGACGCTCATGTGCGAAAGCGTGGGGAGCAAACAGGATTAGATACCTTGGTAGTCCACGCTGTAAACGATGTCGATTTGGGGGTTAACTTTAGGTTGGCGCCCGTAGCTAACGTATA |
| >v06499  GACGTTAACTACAAGAAGAAGCACCGGCTAACTCCGTGCCAGCAGCCGCGGTAATAAGGGAGGGTGCGAGCGTTAATCGGAATAACTGGGAGTAAAGGGCACGCAGGCGGTGACTTAAGTGAGGTGTGAAAGCCCCGGGCTTAACCTGGGAATTGCATTTCATACTGGGTCGCTAGAGTACTTTAGGGAGGGGTAGAATTCCACGTGTAGCGGTGAAATGCGTAGAGATGTGGAGGAATACCGAAGGCGAAGGCAGCCCCTTGGGAATGTACTGACGCTCATGTGCGAAAGCGTGGGGAGCAAACAGGATTAGATACCCTGGTAGTCCACGCTGTAAACGATGTCGATTTGGGGGTTGAACTTTAAGTTTGGCGCCCGTAGCTAACGTGAA |
| >v06644  AACTGTAGCTAATACCGCGTAGAATCGAGAGATGAAAGTGTGGGACCTTCGGGCCACATGCCATAGGATGAGCCCAAGTGGGATTAGGTAGTTGGTGAGGTAAAGGCTCACCAAGCCGACGATCTCTAGCTGGTCTGAGAGGATGACCAGCCACACTGGGACTGAGACACGGCCCAGACTCCTACGGGAGGCAGCAGTGGGGAATATTGCGCAATGGGGGCAACCCTGACGCAGCCATGCCGCGTGAATGAAGAAGGCCTTCGGGTTGTAAAGTTCTTTCGGTAGCGAGGAAGGCATTTAGTTTAATAGACTAGGTGATTGACGTTAACTACAGAAGAAGCACCGGCTAACTCCGTGCCAGCAGCCGCGGTAATACGGAGGGTGCGAGCGTTAATCGGAATAACTGGGCGTAAAGGGCACGCAGGCGGTGACTTAAGTGAGGTGTGAAAGCCCCGGGCTTAACCTGGGAATTGCATTTCATACTGGGTCGCTAGAGTACTTTAGGGAGGGGTAGAATTCCACGTGTAGCGGTGAAATGCGTAGAGATGTGGAGGAATACCGAAGGCGAAGGCAGCCCCTTGGGAATGTACTGACGCTCATGTGCGAAAGCGTGGGGAGCAAACAGGATTAGATACCCTGGTAGTCCACGCTGTAAACGATGTCGATTTGGGGGTTGAGCTTGAGCTGGCGCCCGTAGCTAACGTATA |
| >v09971  GGCAGGCCTTAACACATGCAAGTCGAACGGTAACATAAAGAAGCTTGCTTCTTTGATGACGAGTGGCGGACGGGTGAGTAATGCTTGGGAATCTAGCTTATGGAGGGGGATAACTACGGGAAACTGTAGCTAATACCGCGTAGTATCGAGAGATGAAAGTGTGGGACCTTCGGGCCACATGCCATAGGATGAGCCCAAGTGGGATTAGGTAGTTGGTGAGGTAAAGGCTCACCAAGCCGACGATCTCTAGCTGGTCTGAGAGGATGACCAGCCACACTGGGACTGAGACACGGCCCAGACTCCTACGGGAGGCAGCAGTGGGGAATATTGCGCAATGGGGGCAACCCTGACGCAGCCATGCCGCGTGAATGAAGAAGGCCTTCGGGTTGTAAAGTTCTTTCGGTAGCGAGGAAGGCATTTAGTTTAATAGACTAGGTGATTGACGTTAACTACAGAAGAAGCACCGGCTAACTCCGTGCCAGCAGCCGCGGTAATACGGAGGGTGCGAGCGTTAATCGGAATAACTGGGCGTAAAGGGCACGCAGGCGGTGACTTAAGTGAGGTGTGAAAGCCCCGGGCTTAACCTGGGAATTGCATTTCATACTGGGTCGCTAGAGTACTTTAGGGAGGGGTAGAATTCCACGTGTAGCGGTGAAATGCGTAGAGATGTGGAGGAATACCGAAGGCGAAGGCAGCCCCTTGGGAATGTACTGACGCTCATGTGCGAAAGCGTGGGGAGCAAACAGGATTAGATACCCTGGTAGTCCACGCTGTAAACGATGTCGATTTGGGGGTTGAACTTTAGGTTTGGCGCCCGTAGCTAACGTATA |
| >v10554  CATGCAAGTCGAACGGTAACATAAAGAAGCTTGCTTCTTTGATGACGAGTGGCGGACGGGTGAGTAATGCTTGGGAATCTAGCTTATGGAGGGGGATAACTACGGGAAACTGTAGCTAATACCGCGTAGAATCGAGAGATGAAAGTGTGGGACCTTCGGGCCACATGCCATAGGATGAGCCCAAGTGGGATTAGGTAGTTGGTGAGGTAAAGGCTCACCAAGCCGACGATCTCTAGCTGGTCTGAGAGGATGACCAGCCACACTGGGACTGAGACACGGCCCAGACTCCTACGGGAGGCAGCAGTGGGGAATATTGCGCAATGGGGGCAACCCTGACGCAGCCATGCCGCGTGAATGAAGAAGGCCTTCGGGTTGTAAAGTTCTTTCGGTAGCGAGGAAGGCATTTAGTTTAATAGACTAGGTGATTGACGTTAACTACAGAAGAAGCACCGGCTAACTCCGTGCCAGCAGCCGCGGTAATACGGAGGGTGCGAGCGTTAATCGGAATAACTGGGCGTAAAGGGCACGCAGGCGGTGACTTAAGTGAGGTGTGAAAGCCCCGGGCTTAACCTGGGAATTGCATTTCATACTGGGTCGCTAGAGTACTTTAGGGAGGGGTAGAATTCCACGTGTAGCGGTGAAATGCGTAGAGATGTGGAGGAATACCGAAGGCGAAGGCAGCCCCTTGGGAATGTACTGACGCTCATGTGCGAAAGCGTGGGGAGCAAACAGGATTAGATACCCTGGTAGTCCACGCTGTAAACGATGTCGATTTGGGGGTTGAGCTTTGAGCTTGGCGCCCGTAGCTAACGTATA |
| >v12790  AGTACATTCCCAAGGGGCTGCCTTCGCCTTCGGTATTCCTCCACATCTCTACGCATTTCACCGCTACACGTGGAATTCTACCCCTCCCTAAAGTACTCTAGCGACCCAGTATGAAATGCAATTCCCAGGTTAAGCCCGGGGCTTTCACACCTCACTTAAGTCACCGCCTGCGTGCCCTTTACGCCCAGTTATTCCGATTAACGCTCGCACCCTCCGTATTACCGCGGCTGCTGGCACGGAGTTAGCCGGTGCTTCTTCTGTAGTTAACGTCAATCACCTAGTCTATTAAACTAAATGCCTTCCTCGCTACCGAAAGAACTTTACAACCCGAAGGCCTTCTTCATTCACGCGGCATGGCTGCGTCAGGGTTGCCCCCATTGCGCAATATTCCCCACTGCTGCCTCCCGTAGGAGTCTGGGCCGTGTCTCAGTCCCAGTGTGGCTGGTCATCCTCTCAGACCAGCTAGAGATCGTCGGCTTGGTGAGCC |
| **16S rRNA gene fragments of clinical *H. haemolyticus* strains (n = 3)** |
| >188608  AAGAGTTTGATCCTGGCTCAGATTGAACGCTGGCGGCAGGCTTAACACATGCAAGTCGAACGGTAGCAGGAGAAAGCTTGCTTTCTTGCTGACGAGTGGCGGACGGGTGAGTAATGCTTGGGAATCTGGCTTATGGAGGGGGATAACTACGGGAAACTGTAGCTAATACCGCGTAGTGTCGAGAGACGAAAGTGCGGGACCGCAAGGCCGCATGCCATGAGATGAGCCCAAGTGGGATTAGGTAGTTGGTGGGGTAAAGGCCTACCAAGCCTGCGATCTCTAGCTGGTCTGAGAGGATGGC |
| >188708  GGCAGGCTTAACACATGCAAGTCGAACGGTAGCAGGAGAAAGCTTGCTTTCTTGCTGACGAGTGGCGGACGGGTGAGTAATGCTTGGGAATCTGGCTTATGGAGGGGGATAACTACGGGAAACTGTAGCTAATACCGCGTAGTATCGAAAGATTAAAGTGTGGGACCTTAGGGCCGCATGCCATGAGATGAGCCCAAGTGGGATTAGGTAGTTGGTGGGGTAAAGGCCTACCAAGCCTGCGATCTCTAGCTGGTCTGAGAGGATGGCCAGCCACACTGGAACTGAGACACGGTCCAGACTCCTACGGGAGGCAGCAGTGGGGAATATTGCGCAATGGGGGGAACCCTGACGCAGCCATGCCGCGTGAATGAAGAAGGCCTTCGGGTTGTAAAGTTCTTTCGGTATTGAGGAAGGTTGATGTGTTAATAGTACATCAAATTGACGTTAAATACAGAAGAAGCACCGGCTAACTCCGTGCCAGCAGCCGCGGTAATACGGAGGGTGCGAGCGTTAATCGGAATAACTGGGCGTAAAGGGCACGCAGGCGGTTATTTAAGTGAGGTGTGAAAGCCCCGGGCTTAACCTGGGAATTGCATTTCAGACTGGGTAACTAGAGTACTTTAGGGAGGGGTAGAATTCCACGTGTAGCGGTGAAATGCGTAGAGATGTGGAGGAATACCGAAGGCGAAGGCAGCCCCTTGGGAATGTACTGACGCTCATGTGCGAAAGCGTGGGGAGCAAACAGGATTAGATACCCTGGTAGTCCACGCTGTAAACGCTGTCGATTTGGGGATTGGGCTTAGAGCTTGGTGCCCGTAGCTAACGTGATA |
| >v10510  GCAGTCGAACGGTAGCAGGAGGAAGCTTGCTTTCTTGCTGACGAGTGGCGGACGGGTGAGTAATGCTTGGGAATCTGGCTTATGGAGGGGGATAACTACGGGAAACTGTAGCTAATACCGCGTAATATCGAAAGATTAAAGTGTGGGACCTTCGGGCCACATGCCATAAGATGAGCCCAAGTGGGATTAGGTAGTTGGTGAGGTAAAGGCTCACCAAGCCTGCGATCTCTAGCTGGTCTGAGAGGATGGCCAGCCACACTGGAACTGAGACACGGTCCAGACTCCTACGGGAGGCAGCAGTGGGGAATATTGCGCAATGGGGGGAACCCTGACGCAGCCATGCCGCGTGAATGAAGAAGGCCTTCGGGTTGTAAAGTTCTTTCGGTATTGAGGAAGGGATGTGTGCTAATAGTACACGTCATTGACGTTAAATACAGAAGAAGCACCGGCTAACTCCGTGCCAGCAGCCGCGGTAATACGGAGGGTGCGAGCGTTAATCGGAATAACTGGGCGTAAAGGGCACGCAGGCGGTTATTTAAGTGAGGTGTGAAAGCCCTGGGCTTAACCTGGGAATTGCATTTCAGACTGGGTAACTAGAGTACTTTAGGGAGGGGTAGAATTCCACGTGTAGCGGTGAAATGCGTAGAGATGTGGAGGAATACCGAAGGCGAAGGCAGCCCCTTGGGAATGTACTGACGCTCATGTGCGAAAGCGTGGGGAGCAAACAGGATTAGATACCCTGGTAGTCCACGCTGTAAACGCTGTCGATTTGGGGATTGGGCTTAGAGCTTGGTGCCCGTAGCTAACGTGAT |
| **16S rRNA gene fragment of a *H. parahaemolyticus* strain from a strain collection (n=1)** |
| >v13362  AACAGGAATTAGCTTGCTAATTTGCTGACGAGTGGCGGACGGGTGAGTAATGCTTGGGAATCTGGCTTATGGAGGGGGATAACTACGGGAAACTGTAGCTAATACCGCGTAAAATCTTCGGATTAAAGGGTGGGACTTTCGAGCCACCTGCCATAAGATGAGCCCAAGTGGGATTAGGTAGTTGGTTAGGTAAAGGCTGACCAAGCCGACGATCTCTAGCT |
